# A Peer-to-Peer Suicide Prevention Workshop for Medical Students

**DOI:** 10.15766/mep_2374-8265.11241

**Published:** 2022-04-19

**Authors:** Alyssa Hjelvik, Alyssa Eldridge, Megan Furnari, Hannah Hoeflich, Jason I. Chen, Brandon Roth, Whitney Black

**Affiliations:** 1 Third-Year Medical Student, Oregon Health and Science University School of Medicine; 2 Second-Year Medical Student, Oregon Health and Science University School of Medicine; 3 Assistant Professor, Department of Pediatrics, Oregon Health and Science University School of Medicine; 4 Assistant Professor, Student Health and Wellness, Oregon Health and Science University School of Medicine; 5 Core Investigator, Center to Improve Veteran Involvement in Care, Veterans Affairs Portland Health Care System; Assistant Professor, Department of Psychiatry, Oregon Health and Science University School of Medicine; 6 Research Assistant, Center to Improve Veteran Involvement in Care, Veterans Affairs Portland Health Care System, Portland Veterans Affairs Research Foundation, and Department of Psychiatry, Oregon Health and Science University School of Medicine; 7 Associate Professor, Department of Psychiatry, Oregon Health and Science University School of Medicine

**Keywords:** Suicide Prevention, Peer-to-Peer, Role-Play, Well-Being/Mental Health

## Abstract

**Introduction:**

An estimated 11% of medical students experience suicidal ideation during medical school. Many medical schools teach students how to intervene on behalf of patients experiencing suicidal ideation, but no curriculum in *MedEdPORTAL* teaches students how to intervene on behalf of peers.

**Methods:**

The authors designed, implemented, and evaluated a 2-hour workshop to equip medical students with skills and resources to intervene on behalf of a peer in crisis. This workshop comprised a peer-led didactic session and small-group sessions with role-plays and a guided debrief. The resource included a slide deck for the didactic session, a facilitator guide for the small-group session, a student handout with role-plays and self-evaluation questions, and the pre-/postsurvey.

**Results:**

This workshop was conducted with cohorts of first- and second-year medical students (*n* = 273) in October and November 2019. Pre-/postsurveys showed the greatest improvements in suicide prevention knowledge (self-rated) and the confidence in and likelihood of asking peers about suicide.

**Discussion:**

Student feedback indicated that the most valuable parts of the workshop were the peer-led nature of the didactic session, the perspective of a peer's lived experience, and the role-plays. Opportunities for improvement included the scheduling of the session, the potentially triggering nature of the role-play exercises, and the importance of enabling students to opt out discreetly. A version of this workshop is now a permanent part of the first-year curriculum at our institution.

## Educational Objectives

By the end of this activity, learners will be able to:

1. Restate the prevalence of mental illness and suicidal ideation among medical trainees.
2. List modifiable and nonmodifiable risk factors and warning signs associated with suicide as a first step toward understanding how to provide resources to a peer in need.
3. Describe the importance of asking directly about suicidal thoughts, intentions, and access to lethal means if a peer is showing warning signs.
4. Demonstrate confidence in deploying skills to assist a suicidal peer.
5. Explain how to direct a peer to routine care services, crisis care services, and local and national suicide prevention resources.

## Introduction

Rates of depression and suicidal ideation may be higher among medical students than age-matched peers. A 2016 meta-analysis of 195 studies reported that about 27% of medical students had experienced depression during school and that 11% had experienced suicidal ideation (*n* = 129,123 medical students from 47 countries).^1^ By contrast, among all US adults ages 20–39, the Centers for Disease Control estimated a prevalence of depression of 8% from 2013 to 2016.^2^ Similarly, in a systematic review of 40 articles, Dyrbye and colleagues found that medical students consistently experienced a higher level of psychological distress (including depression, anxiety, and burnout) than age-matched peers and the general population.^3^ Medical students start their training with mental health backgrounds that are similar to age-matched peers,^2,4^ so for many, mental illness takes hold for the first time during medical school. Medical trainees may be more susceptible to suicidal ideation because of burnout, financial debt, social isolation, and an excessive sense of responsibility for patients’ health outcomes.^5,6^

Unfortunately, medical students tend to be reluctant to seek help for symptoms of depression or burnout.^7–9^ In this regard, medical students behave similarly to other young adult students. An online survey of over 26,000 undergraduate and graduate students at 70 colleges and universities in the US found that when students choose to reach out, most opt to tell a peer first.^10^ Given this information, it is critical to equip students with meaningful skills to intervene on behalf of a peer in crisis.

High schools and colleges around the country have embraced the principle of peer-to-peer suicide prevention.^11–13^ For example, in an open pilot trial at a university campus on the US mid-Atlantic coast, 231 college students received a 1-hour suicide prevention gatekeeper training. The program was associated with increased suicide prevention knowledge, and at the 3-month follow-up, the workshop significantly increased both the number of students who identified a suicidal peer and the number of students who referred at least one suicidal peer to mental health care.^14^ This evidence suggests that even a brief onetime gatekeeper workshop for students may be a promising way to reach students who otherwise are unlikely to reach out.

Despite the popularity of peer-based approaches in many academic settings, few publications have investigated strategies for making medical trainees more resilient to mental health crises. In medicine, most discussions of suicide focus on patients rather than providers. In *MedEdPORTAL,* a keyword search for *suicide* yields more than 40 publications regarding suicide risk assessments and management for patients. By contrast, only four publications pertain to medical trainees/providers—three of which mention suicide only in passing—in the context of resident and physician wellness.^15–18^ Only one curriculum in *MedEdPORTAL* focuses on depression and suicide among medical trainees (specifically, interns, residents, and fellows). That curriculum's 60-minute video and discussion guide represent an important step toward destigmatizing mental illness in this population, but the curriculum does not train participants to intervene on behalf of a peer, nor is it tailored for undergraduate medical students.^18^

In recognition of the lack of resources for suicide prevention among medical students, we developed an initial workshop in May 2019. Seven first-year medical students signed up for a 2-hour workshop, divided between a Talk Saves Lives presentation from a representative of the American Foundation for Suicide Prevention (AFSP)^19^ and supervised role-plays with a debrief.^20^ Based on a pre-/postsurvey, we modified the curriculum for delivery to a larger medical student audience, and we used a peer-to-peer approach to make the content seem more relevant and accessible to students. The medical school's first- and second-year classes participated in the revised workshop in the fall of 2019.

## Methods

### Participants

Our team consisted of two second-year medical students, the director of medical student wellness and leadership development, and faculty from the department of psychiatry and student health and wellness. The implemented workshop included a 45-minute didactic session followed by 1 hour of role-plays and discussion in small groups. The didactic session's structure and some of the slides were adapted from the AFSP Talk Saves Lives materials^19^ with permission, and citations were included on the adapted slides; otherwise, we created the materials. This publication includes the PowerPoint slides for the didactic session (Appendix A), a student guide containing the role-plays and self-evaluation prompts (Appendix B), and a facilitator guide for the small-group sessions (Appendix C).

Our team worked collaboratively to develop the slide deck, facilitation guide, and sample role-plays. UME leadership reviewed and approved the materials. Small-group faculty facilitators received access to both the slides and faculty guide and attended a 1-hour orientation in the weeks before the live workshop.

Medical students did not require any prerequisite knowledge to attend the workshop. A week before the workshop, an email notified students of the sensitive content and advised them that they could opt out; they would not be evaluated on attendance or participation.

### Procedures

On the day of each workshop, medical students gathered in a large classroom at the medical school for the didactic session, led by the two peer facilitators. The didactic session began with a welcome, introductions, distribution of a card listing local and institution-specific mental health resources, and completion of the presurvey (Appendix D; 5 minutes maximum). During the lecture, the peer facilitators discussed mental illness among medical trainees, risk factors, warning signs, common myths about suicide, principles for approaching conversations about suicidal ideation, and local and institution-specific resources, with commentary from a peer with lived experience. The didactic session was structured around a PowerPoint slide deck (Appendix B; 35 minutes maximum). The peer facilitators concluded the didactic session with a role-play demonstration to prepare participants for the subsequent small-group role-play exercises. Students were notified of their small-group assignments and advised that they could opt out of the small-group session without consequences.

Following the didactic session, students had 15 minutes to take a break and transition to their small-group locations. Each small group consisted of approximately 10 students with two faculty facilitators. Students were asked to pair up for the role-play exercises (Appendix B; 8–10 minutes per role-play). After completing each role-play, students used self-evaluation prompts to debrief within their pairs (Appendix B; 5 minutes per role-play). Then, the full small group reconvened for a more comprehensive, facilitated debrief (Appendix C; 25 minutes maximum) and a brainstorm of take-home points. Facilitators had a final 5 minutes to wrap up the session and administer the postworkshop survey (Appendix D).

At several times during the workshop, a facilitator informed students that a psychologist from the organization's Office of Student Health and Wellness was available on site for psychological support as needed during and after the workshop.

### Measures

To assess the impact of the workshop, we developed a self-report pre-/postsurvey using Ajzen's theory of planned behavior as a measurement framework because its constructs have been shown to predict actual behavior.^21,22^ We defined three goal behaviors of our workshop (identifying peers at risk of suicide, directly asking at-risk peers about suicide, and making referrals to appropriate resources) and then developed evaluations of attitudes, confidence, and intentions to engage in the goal behaviors (Table 1 includes the prompts). The presurvey and postsurvey included identical sets of yes/no statements and 5-point Likert scales (1 = *strongly disagree,* 5 = *strongly agree*). The postsurvey also asked free-response questions about the most and least helpful portions of the workshop, additional resources requested, and what information would facilitate increased confidence in peer-to-peer suicide prevention (Table 2 includes the prompts). The survey was approved by our organization's institutional review board.

**Table 1.**
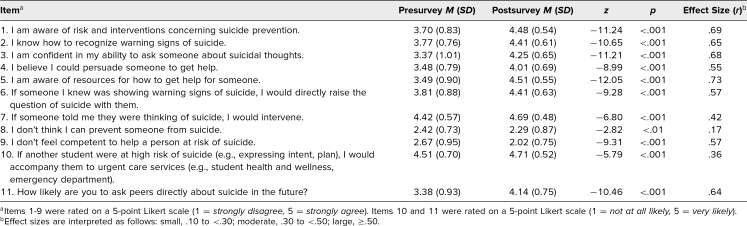
Pre- and Postsurvey Mean Ratings and Effect Sizes by Item (*N* = 269)

**Table 2.**
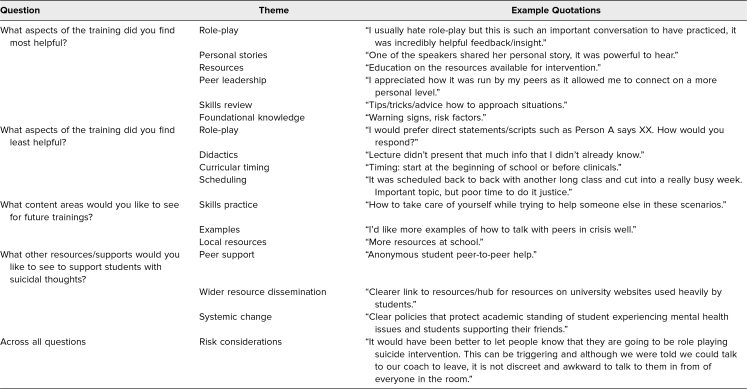
Themes and Representative Quotes From Medical Student Responses to Open-Ended Postsurvey Questions

Students received paper copies of the pre-/postsurvey as they entered the didactic session. We asked them to complete the presurvey before the start of the workshop and the postsurvey at the end of the small-group session. Participation was voluntary and anonymous; the survey did not ask for a student's name and did not collect demographic information.

## Results

A total of 273 first- and second-year medical students participated in the presurvey, and 269 also completed the postsurvey. All the ordinal items were screened for normality (e.g., skew) and other test assumptions. Table 1 reports the means and results of the Wilcoxon signed rank test. We used the *r* correlation coefficient to calculate effect sizes.

Generally, students reported that the workshop was valuable, and they showed significant improvement on all 11 survey items. Students showed the largest improvements in their perceived suicide prevention knowledge, confidence in asking about suicide, awareness of resources, likelihood of asking peers directly about suicide, and belief that they could persuade a peer to get help. We saw moderate increases in the likelihood of intervening if someone mentioned they were thinking of suicide and in the likelihood of seeking urgent care services for high-risk peers. We observed a smaller improvement in the perception that suicide is preventable.

For the free-response postsurvey questions, two authors (Jason I. Chen and Brandon Roth) conducted thematic analysis.^23^ Specifically, they independently reviewed all responses to develop potential themes within each question and then met to resolve discrepancies in the identified themes via joint consensus.^24,25^ Table 2 shows the observed themes and representative quotes for each question.

## Discussion

Despite the recognized risk of suicide among medical students, residents, and physicians, the medical field has inadequate training, education, and resources for suicide prevention. To our knowledge, this workshop is the first in *MedEdPORTAL* to provide peer-to-peer suicide prevention training to medical students, and it also is the first formal peer suicide prevention education in our medical school curriculum.

This publication describes the implementation and initial results from the fall 2019 workshops at our institution. Our pre/post quantitative analyses showed significant increases in students’ confidence in asking about suicide, likelihood of using direct language to ask a peer about suicide, and awareness of resources available at our institution, locally, and nationally. In our qualitative analyses, many students found the small-group discussions and role-plays to be effective for learning to apply concepts from the didactic session to actual conversations. The results suggest that a brief peer-led workshop can equip students to identify and refer at-risk peers to appropriate resources.

The workshop received the most consistently positive feedback for the peer-to-peer element (i.e., the fact that two students designed and delivered the didactic session). Notably, one student had lived experience with suicidal ideation and intent, and she integrated narratives about her own experience to offer insight into a state of mind that might seem illogical and even frightening to others. Student feedback indicated that both components—the inclusion of lived experience and the simple fact that the presentation was delivered by peers—helped establish rapport, build empathy, and increase comfort with the concept of intervening on behalf of peers with suicidal ideation.

We received actionable feedback on three major themes. First, it is important to consider the timing and scheduling of the workshop in relation to other demands and courses. For instance, our presentation for second-year students occurred just weeks after the students received a lecture on clinical suicide prevention, so some of the foundational materials (e.g., risk factors and warning signs) were redundant. Meanwhile, our presentation for first-year students was scheduled back-to-back with another 2-hour small-group discussion on an emotionally intense topic.

Second, while role-plays have been found to increase self-efficacy and crisis-related knowledge,^26^ they may also present challenges depending on the cultural background, race, ethnicity, gender identity, and sexual orientation of the participants. For example, suicide in some populations may be viewed as an outcome attributed to historical and current oppression in addition to the suffering that injustice perpetuates, rather than as an individual reacting to psychological distress. To ensure that the curriculum is tailored to the needs of the student population, students ideally should help create and deliver the workshop.

Third, the sensitive nature of this topic warrants a trauma-informed approach. Although we believe that suicide is an important topic for all medical trainees, it certainly is possible to do more harm than good, especially for students with recent or personal experience with suicide. We informed students that they could opt out of the workshop, but some provided feedback that they were unaware of this option, and others wished that they could have learned about the topic in other ways. We recommend offering workshop alternatives (e.g., asynchronous learning) and communicating options via multiple channels (e.g., an email, post on an internal website, and announcement at the start of the workshop).

Still, the topic may be difficult even for those who choose to participate (or do not opt out), including those with no personal experience with suicide. Small-group facilitators must be comfortable discussing the topic and should receive basic suicide prevention training in advance. We also recommend offering professional resources (e.g., counseling drop-in hours) during and after the session as the topic may be triggering regardless of the precautions taken. Similarly, any students who help deliver the workshop should be provided with faculty support given the intensely vulnerable nature of this undertaking.

Limitations of our workshop include the onetime format and the time and resources required for high-quality small-group sessions. We also cannot comment on sustainability, as these were the first large-scale workshops at our institution. In addition, our evaluation method did not allow us to test whether the workshops led to relevant behavioral changes (e.g., referring at-risk peers to counseling) or long-term changes in attitudes and confidence. This publication represents a first step toward developing best practices for training medical students to aid peers experiencing suicidal ideation, and we hope others will refine this approach and publish more robust empirical evidence.

Our results suggest that near-peer leadership and active small-group sessions are effective components for building resilience to suicide in medical trainee communities. At the same time, we wish to emphasize that the education needed for peer suicide prevention is complex. We advocate for a graduated curriculum that is integrated into the course of education, training, and practice. This workshop has been designed to be a formative experience that prepares medical trainees for ongoing conversations about mental illness and suicide by providing basic knowledge, resources, and an opportunity to approach the topic from a compassionate rather than stigmatized perspective. Providing peer suicide prevention training early in medical school may have a long-term impact on the ability of medical students, residents, and faculty to recognize at-risk peers and connect them with appropriate resources. We hope other institutions will join us in making this a routine part of medical education.

## Appendices


Didactic Slide Deck.pptxStudent Guide.docxFaculty Facilitation Guide.docxPre- and Postsurveys.docx
*All appendices are peer reviewed as integral parts of the Original Publication.*
