# Supplementary material for: A Peer-to-Peer Suicide Prevention Workshop for Medical Students
Source: MedEdPORTAL. 2022 Apr 19;18:11241. doi: 10.15766/mep_2374-8265.11241 (PMC9016109; doi:10.15766/mep_2374-8265.11241)
Supplement: Supplementary file 1 — Didactic Slide Deck.pptxStudent Guide.docxFaculty Facilitation Guide.docxPre- and Postsurveys.docx [file mep_2374-8265.11241-s001.zip › B. Student Guide.docx]

**AGENDA for Role-Play (total time 10 minutes):**

- - Student pairs should take no more than 8 minutes to act out the scenario. This should be enough time for the intervener to:
    - Open a conversation and express concern about their peer.
    - Briefly explore their peer’s situation to whatever extent the peer wishes to share—remember, the intervener is not providing therapy or attempting to “solve” anything.
    - Ask directly about suicidal thoughts, plans, and/or intentions.
    - Express concern and offer support via access to professional resources and/or securing lethal means.

**Self-evaluation Questions to complete after role-play complete (discuss with your partner):**

- Did you/they recognize the warning signs?
- Did you/they show care and concern and/or offer help and support?
- Did you/they ask directly about suicide?
- Did you/they listen without judgment?
- Did you/they know their role as a peer supporter?  These conversations look different with patients versus peers.
- Can you/they connect the student in need to the appropriate resources, counseling, and clinician who can do a risk assessment?

**Guide for the person doing the intervention:**

**DO:**

- Be yourself. Let the person know you care and that he/she/they is not alone. The right words are often unimportant. If you are concerned, your voice and manner will show it.
- Listen. Let the suicidal person unload despair and vent anger. No matter how negative the conversation seems, the fact that it is taking place is a positive sign.
- Be sympathetic, non-judgmental, patient, calm, and accepting. Your friend or family member is doing the right thing by talking about his/her/their feelings.
- Offer hope. Reassure the person that help is available and that the suicidal feelings are temporary. Let the person know that his/her/their life is important to you.
- Take the person seriously. If the person says things like, “I’m so depressed, I can’t go on,” ask the question: “Are you having thoughts of suicide?” You are not putting ideas in their head; you are showing that you are concerned, that you take them seriously, and that it is okay for them to share their pain with you.

**DON’T:**

- Argue with the suicidal person. Avoid saying things like: “You have so much to live for,” “Your suicide will hurt your family,” or “Look on the bright side.”
- Act shocked, lecture on the value of life, or say that suicide is wrong.
- Promise confidentiality; instead, refuse to be sworn to secrecy. A life is at stake, and you may need to speak to a mental health professional in order to keep the suicidal person safe. If you promise to keep your discussions secret, you may have to break your word.
- Offer ways to fix their problems, give advice, or make them feel like they have to justify their suicidal feelings. It is not about how bad the problem is, but how badly it is hurting your friend or loved one.
- Blame yourself. You cannot “fix” someone’s depression. Your peer’s happiness, or lack thereof, is not your responsibility.

**Role Play #1: Perspective of the Peer with Suicidal Thoughts**

- You are in the spring of your third year.
- Your partner of several years died in a car accident one month ago. You were not involved in the accident.
- The week after the accident, you were scheduled to start a rural family medicine continuity rotation (2 months long). You decided to keep this rotation instead of requesting time off because you hope to go into rural family medicine, and you were afraid that you would not have another opportunity like this one before applying for residency.
- So, for the past three weeks, you have been at a rural location in Oregon, where you do not know anyone other than the healthcare providers you have met since you arrived.
- Your support network consists of the following:
  - You have a decent overall relationship with your family (lives out of state). Unfortunately, they were not very supportive of your relationship, so you feel unable to reach out to them as you grieve your partner.
  - You have a strong support network back in Portland, but even though you were not involved in the accident, you have developed a fear of driving and cannot bring yourself to come back to Portland for a weekend though you are able to bike to the clinic and walk around town.
  - Most of your friends in Portland are also third-year medical students, so they are crazy busy with their own rotations. Many reached out, especially at first, but you felt bad about the intensity of your emotional needs and unpredictability of your mood… especially given that your peers are stressed with their own school/career/life challenges.
  - Other non-med-school friends have reached out as well, but mostly to express concern that you should be taking time off. You got frustrated and angry on several occasions that no one outside of medicine seemed to understand how important this rotation is to your career.
- So far, you have been able to perform satisfactorily on the rotation. You actually feel okay during the day, when you are busy in the clinic, but your mood and sense of stability deteriorate as soon as you go home at night. (You are renting an accessory dwelling unit and do not really know the owners of the main property, so you essentially live alone.)
- Your mental state and ability to cope (or not) are the following:
  - When you get home in the evenings, your biggest problem is anxiety—without anything to distract you, anxiety mixes into your cycling emotions of sadness, anger, guilt, and hopelessness. It consumes you for hours until you are able to put yourself to bed… and even then, it is hard to sleep.
  - You have realized that you feel less anxious and have less trouble sleeping if you drink alcohol. You started with a glass of wine with dinner; now you drink closer to a bottle each night. You wake up sober and feel like this coping strategy is not hurting your performance in clinic. It feels sustainable, at least until the end of the rotation.
  - When your mood is at its worst, you feel utterly paralyzed with hopelessness about the future. It is hard to imagine life without your partner, and it is also hard to imagine asking friends for enough support to recover.
  - Especially once you have had some alcohol, your mind sometimes wanders to the Tylenol bottle in the bathroom. Sometimes, you feel consumed by an impulse to take as many pills as you can. By the morning, you no longer think this is a good idea, but it scares you that your brain often goes to this place.
- Although you have generally been ignoring texts and calls from most friends, you agreed to talk with one friend this evening. They were supportive of your decision to participate in the rotation after the accident, so you feel more open to talking with them about how you are doing.

**Role Play #1: Perspective of the Peer Who Wishes to Intervene**

- You are in the spring of your third year.
- One month ago, one of your friends lost their partner in a car accident. Your friend was not involved in the accident.
- A week after the accident, your friend was scheduled to start a rural family medicine rotation (2 months long). The timing was terrible because this should have been the high point of their rotations—they have always wanted to do rural family medicine and felt like this was exactly the opportunity they needed.
- You have been reasonably close to this friend since your first year of medical school, but you are not among their *closest* friends. You were not one of the people who your friend initially called for support when their partner died.
- You know that some of your peers tried to talk your friend into taking time off, but your friend decided to go anyway.
- When you talked with your friend right before they left town, they expressed confidence that they were making a smart career choice and also optimism that staying busy with a rotation was a good coping strategy. They were frustrated that no one seemed to respect how important this rotation was for them.
- Since your friend started the rotation, you have talked only a couple of times, and both times were during evenings of the first week. Your friend sounded pretty subdued but tried to put a positive spin on things and reassure you.
- Since then, you have sent a few texts and left a voicemail, but you have not heard back. You just talked with a couple of mutual friends and realized that no one had been in touch with your friend for at least a week, which was pretty concerning to all of you. You sent a more insistent text and your friend responded and agreed to chat this evening.

**Role Play #2: Perspective of the Peer with Suicidal Thoughts**

- You are in the winter of your third year. You have completed five of your core rotations, including the ones you were most excited about, but you did not enjoy them the way you expected to.
- In fact, you have not really enjoyed *any* of your core or elective rotations. You have been struggling to connect with patients emotionally, you have not enjoyed the diagnostic process like you were expecting to, and you have not felt excited about the procedures you used to look forward to.
- For several months now, you have felt increasingly like you made a terrible mistake by going to medical school. You find yourself resenting the work and the patients, and then you feel guilty about this resentment; you feel angry at yourself for the choices you have made, and angry at others for pushing you down this path. Most of all, you feel trapped.
- You have sought advice from a few people:
  - A financial advisor recommended that you earn your MD, and then, instead of going to residency, pursue something lucrative to address your debt. You are an out of state student, so that does not help, and additionally, none of those “lucrative” options sounded remotely appealing to you.
  - Your parents were shocked and disappointed to hear that you wanted to leave medicine. Though they then tried to offer support, their initial reaction was strong enough to deter you from further conversation.
  - A faculty mentor was sympathetic and perceptive of your situation a few months ago. Unfortunately, they are on parental leave until the summer.
  - Several peers and friends have been supportive to varying degrees. However, you get annoyed when they try to cheer you up and promise you that you will “find your calling” and that you just have not stumbled upon it yet. They do not seem to get how disconnected you feel from *all* patients and *all* medicine… it is *not* about the specialty anymore! Because of these previous encounters, you have not pursued one of these conversations for a while.
- Your mental state and ability to cope (or not) are the following:
  - You recognize that you are depressed. You struggled with depression in high school and took medication for it, but you felt stable without medication ever since then. Now, you are aware of your depression but you are skeptical that treating it would fix anything—you feel that you are depressed *because* you have made a terrible career choice, so depression is not the root of the problem.
  - On top of your guilt and helplessness regarding your career, you feel terrible that your “bad attitude” (as you think of it) is affecting people you care about. You know your parents are disappointed and worried; you know your friends are only trying to help, and you have responded with anger. It feels safer to isolate yourself—at least that way, you know you are not hurting anyone else.
  - Until a few months ago, you had robust independent coping strategies (e.g., going to the gym, walking your roommate’s dog, sitting outside and reading), but you have not had the energy to do any of that recently.
  - You discovered that you *do* feel better (temporarily) if you hurt yourself a little—a small cut or burn, nothing life-threatening, but when you are in your darkest places, often on Sunday evenings, you imagine using the scalpel you took from the hospital to end your life. You are not sure if you want to, but the thought makes you feel calmer, somehow.
- On your current rotation, a peer who you do not know particularly well has been trying to check in with you, and you have avoided talking much. Today, you got out earlier than usual, and your peer asked to grab dinner with you. You agreed.

**Role Play #2: Perspective of the Peer Who Wishes to Intervene**

- You are in the winter of your third year.
- You have become increasingly worried about a peer who is on your current rotation. You do not know them particularly well; you participated in one of the same interest groups during the pre-clinical years but you had not maintained a relationship until starting this rotation together.
- Even though you never knew this peer well, you feel that something has changed. They always seem exhausted and disconnected now, and they seem super sensitive to even the gentlest feedback. You have tried to connect with them in small moments between patients, but they always redirect the conversation.
- Since your gut instinct told you something was wrong, you reached out to a couple of your peer’s friends. They told you that this peer has struggled to find a specialty they like, and they have become depressed about it. You asked your peer’s friends if they had checked in recently to make sure your peer was okay, and they admitted that they had been fairly distant—their most recent conversations with this peer had ended with anger, as the peer seemed to feel that no one understood their situation.
- You decide that your peer’s behavior and apparent isolation is concerning enough to warrant a conversation, and you convince them to grab dinner with you when you get out early one evening.
