# Supplementary material for: A Peer-to-Peer Suicide Prevention Workshop for Medical Students
Source: MedEdPORTAL. 2022 Apr 19;18:11241. doi: 10.15766/mep_2374-8265.11241 (PMC9016109; doi:10.15766/mep_2374-8265.11241)
Supplement: Supplementary file 1 — Didactic Slide Deck.pptxStudent Guide.docxFaculty Facilitation Guide.docxPre- and Postsurveys.docx [file mep_2374-8265.11241-s001.zip › C. Faculty Facilitation Guide.docx]

**FACULTY FACILITATION GUIDE FOR PEER-TO-PEER SUICIDE PREVENTION**

[Blinded]

**About**

[Blinded]

**Structure**

**1-1:45 PM** will involve a didactic presentation on risk factors, warning signs, and common myths about suicide, as well as an overview of intervention techniques and a demonstration of those techniques. A wallet-sized resource card will be given to all medical students with relevant phone numbers and resources at [blinded] as well as throughout [blinded]; this will be a reference for the second half of the workshop and moving forward at [blinded]. Faculty coaches are encouraged to attend the presentation but will not have any responsibilities during this portion of the workshop.

**1:45-3 PM** will involve role-play exercises and debriefing in small groups, assigned by coaching cohort. Faculty coaches will guide medical students through these activities, as explained in this guide. Coaching cohorts will be paired up to provide more faculty support. **Coaches should pay close attention to time, announcing transitions to students based on the below.** **Coaches can also move between pairs to observe, listen, and take notes on how things are going during scenarios. [Blinded] will be moving around between rooms to check-in with coaches and observe as needed. They will be a resource to you all.**

- **1:45 –1:50 PM Transition to Small Groups**
- **1:50-2:30 PM Role-Plays**
  - You will be provided with scenarios that are relevant to the medical school experience. Each scenario is described from two perspectives: the student who is having thoughts of suicide and the student who is concerned and wishes to intervene.
  - Students should pair up and decide who will play which role for the first scenario; they will switch for the second. If there is an odd number and thus a group of three, two students can try intervening together so that everyone gets practice.
  - **1:50-1:55 PM** Each student should read the scenario from the perspective of their designated role ONLY. They can also review the Dos/Don’ts if they are the student doing the intervention.
  - **1:55-2:05 PM** Student pairs should take no more than 8 minutes to act out the scenario. This should be enough time for the intervener to (below is included in student handout):
    - Open a conversation and express concern about their peer.
    - Briefly explore their peer’s situation to whatever extent the peer wishes to share—remember, the intervener is not providing therapy or attempting to “solve” anything.
    - Ask directly about suicidal thoughts, plans, and/or intentions.
    - Express concern and offer support via access to professional resources and/or securing lethal means.
  - **2:05- 2:10 PM** As students finish, they refer to the self-evaluation questions (included in the student handout, see below) and can reflect on their conversation.
  - **2:10-2:15 PM** Students move on to the second role-play scenario and review new role descriptions. Review Dos/Don’ts list.
  - **2:15-2:25 PM** Announce that it is time to start the second scenario if they have not already.
  - **2:25-2:30 PM** Students refer to self-evaluation questions and reflect.
- **2:30-2:45 PM Small Group Debrief**
  - Bring the students in your cohort back together, ideally in a circle of chairs without tables.
  - Use the following prompts to discuss outcomes from the role-plays and troubleshoot together. You do not have to cover all of these questions as long as the conversation is productive, but this may be a useful structure if students are reluctant to share.
    - What was most difficult, surprising, or uncomfortable about the role-plays?
    - Was it hard to ask directly about suicide? (Perhaps students who found it easier can share how they asked.)
    - Once you learned that your peer was having thoughts of suicide, what specific support did you offer? Did you feel confident in that choice?
    - What uncertainties came up at any point in the process?
    - What additional resources, training, and/or practice would you need to feel really confident intervening on behalf of a peer?
  - If a question comes up that you cannot answer, email it to [blinded]; you can send the response to your coaching cohort in a follow-up email.
- **2:45 PM – 3 PM Feedback and Close**
  - **2:45- 2:55 PM**
    - Ask for “plus/delta” feedback from the students and write their responses on the white board. Plus is something they liked and delta is something they would change to improve the experience next time.
    - If there is time, ask each student to share one take-home point that they will remember from the workshop. (It could be a particular “do” or “don’t,” a dispelled myth about suicide, a useful resource, etc.).
  - **Email a photo of the “plus/delta” and “take-homes” to Dr. [blinded].**
  - **2:55- 3 PM** Complete the post-survey, which students will have with them and coaches collect and leave in the rooms. Students can leave when they are finished. Students can take the role-play and resources packet home with them.

**Expert Recommendations**

*The following suggestions may be helpful for you as faculty to guide students while observing role-plays.*

**Self Evaluation Questions/Faculty Observation Questions:** Look for the following in the student who is playing the support role/intervening.

- Did you/they recognize the warning signs?
- Did you/they show care and concern and/or offer help and support?
- Did you/they ask directly about suicide?
- Did you/they listen without judgment?
- Did you/they know their role as a peer supporter?  These conversations look different with patients versus peers.
- Can you/they connect the student in need to the appropriate resources, counseling, and clinician who can do a risk assessment?

**DO:**

- Be yourself. Let the person know you care and that he/she/they is not alone. The right words are often unimportant. If you are concerned, your voice and manner will show it.
- Listen. Let the suicidal person unload despair and vent anger. No matter how negative the conversation seems, the fact that it is taking place is a positive sign.
- Be sympathetic, non-judgmental, patient, calm, and accepting. Your friend or family member is doing the right thing by talking about his/her/their feelings.
- Offer hope. Reassure the person that help is available and that the suicidal feelings are temporary. Let the person know that his/her/their life is important to you.
- Take the person seriously. If the person says things like, “I’m so depressed, I can’t go on,” ask the question: “Are you having thoughts of suicide?” You are not putting ideas in their head; you are showing that you are concerned, that you take them seriously, and that it is okay for them to share their pain with you.

**DON’T:**

- Argue with the suicidal person. Avoid saying things like: “You have so much to live for,” “Your suicide will hurt your family,” or “Look on the bright side.”
- Act shocked, lecture on the value of life, or say that suicide is wrong.
- Promise confidentiality; instead, refuse to be sworn to secrecy. A life is at stake, and you may need to speak to a mental health professional in order to keep the suicidal person safe. If you promise to keep your discussions secret, you may have to break your word.
- Offer ways to fix their problems, give advice, or make them feel like they have to justify their suicidal feelings. It is not about how bad the problem is, but how badly it is hurting your friend or loved one.
- Blame yourself. You cannot “fix” someone’s depression. Your peer’s happiness, or lack thereof, is not your responsibility.
