# Supplementary material for: A Peer-to-Peer Suicide Prevention Workshop for Medical Students
Source: MedEdPORTAL. 2022 Apr 19;18:11241. doi: 10.15766/mep_2374-8265.11241 (PMC9016109; doi:10.15766/mep_2374-8265.11241)
Supplement: Supplementary file 1 — Didactic Slide Deck.pptxStudent Guide.docxFaculty Facilitation Guide.docxPre- and Postsurveys.docx [file mep_2374-8265.11241-s001.zip › A. Didactic Slide Deck.pptx]

## Slide 1
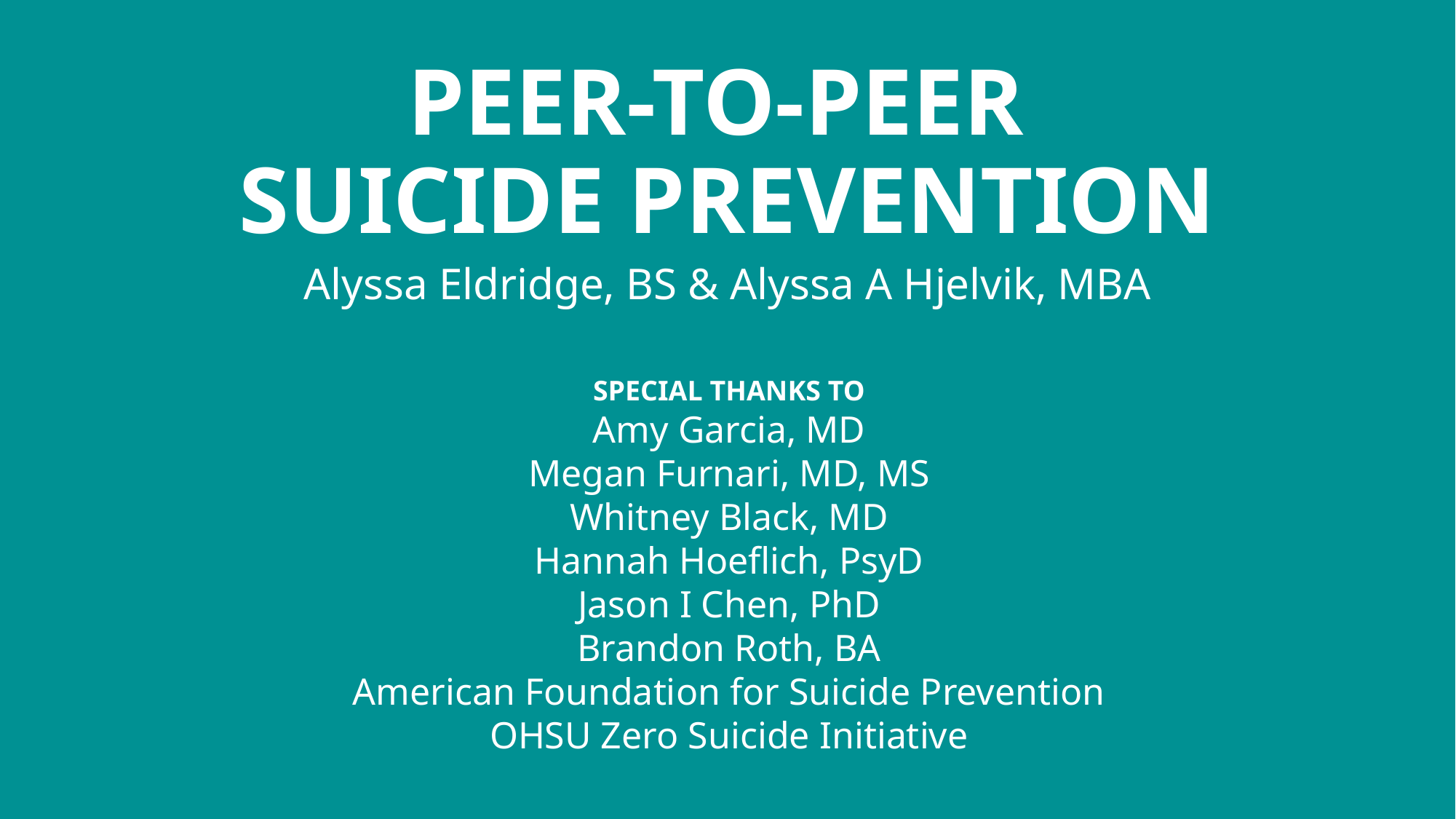

# PEER-TO-PEER SUICIDE PREVENTION
Alyssa Eldridge, BS & Alyssa A Hjelvik, MBA
SPECIAL THANKS TO
Amy Garcia, MD
Megan Furnari, MD, MS
Whitney Black, MD
Hannah Hoeflich, PsyD
Jason I Chen, PhD
Brandon Roth, BA
American Foundation for Suicide Prevention
OHSU Zero Suicide Initiative

## Slide 2
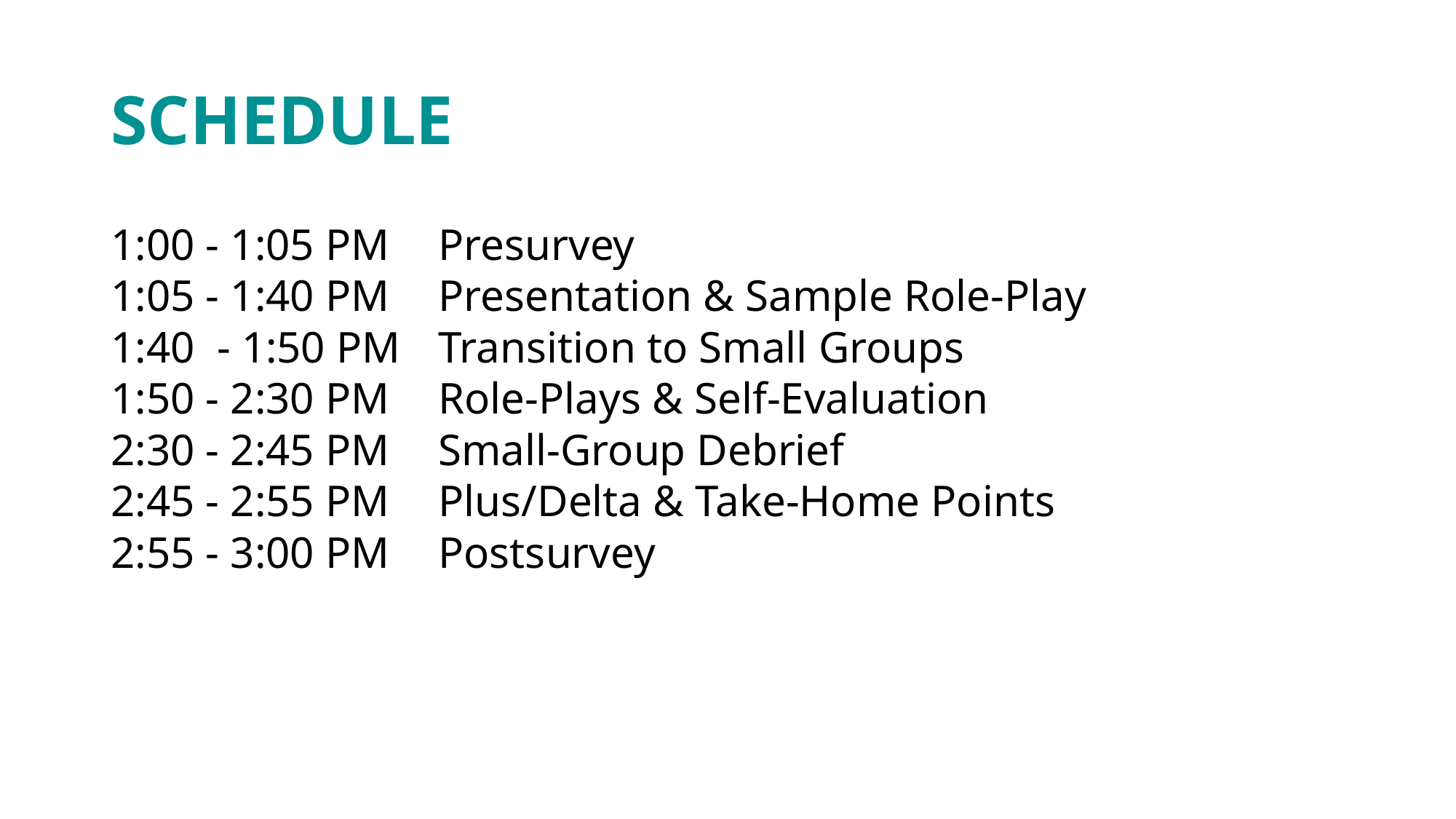

# SCHEDULE
1:00 - 1:05 PM	Presurvey
1:05 - 1:40 PM	Presentation & Sample Role-Play
1:40 - 1:50 PM	Transition to Small Groups
1:50 - 2:30 PM	Role-Plays & Self-Evaluation
2:30 - 2:45 PM	Small-Group Debrief
2:45 - 2:55 PM	Plus/Delta & Take-Home Points
2:55 - 3:00 PM	Postsurvey

## Slide 3
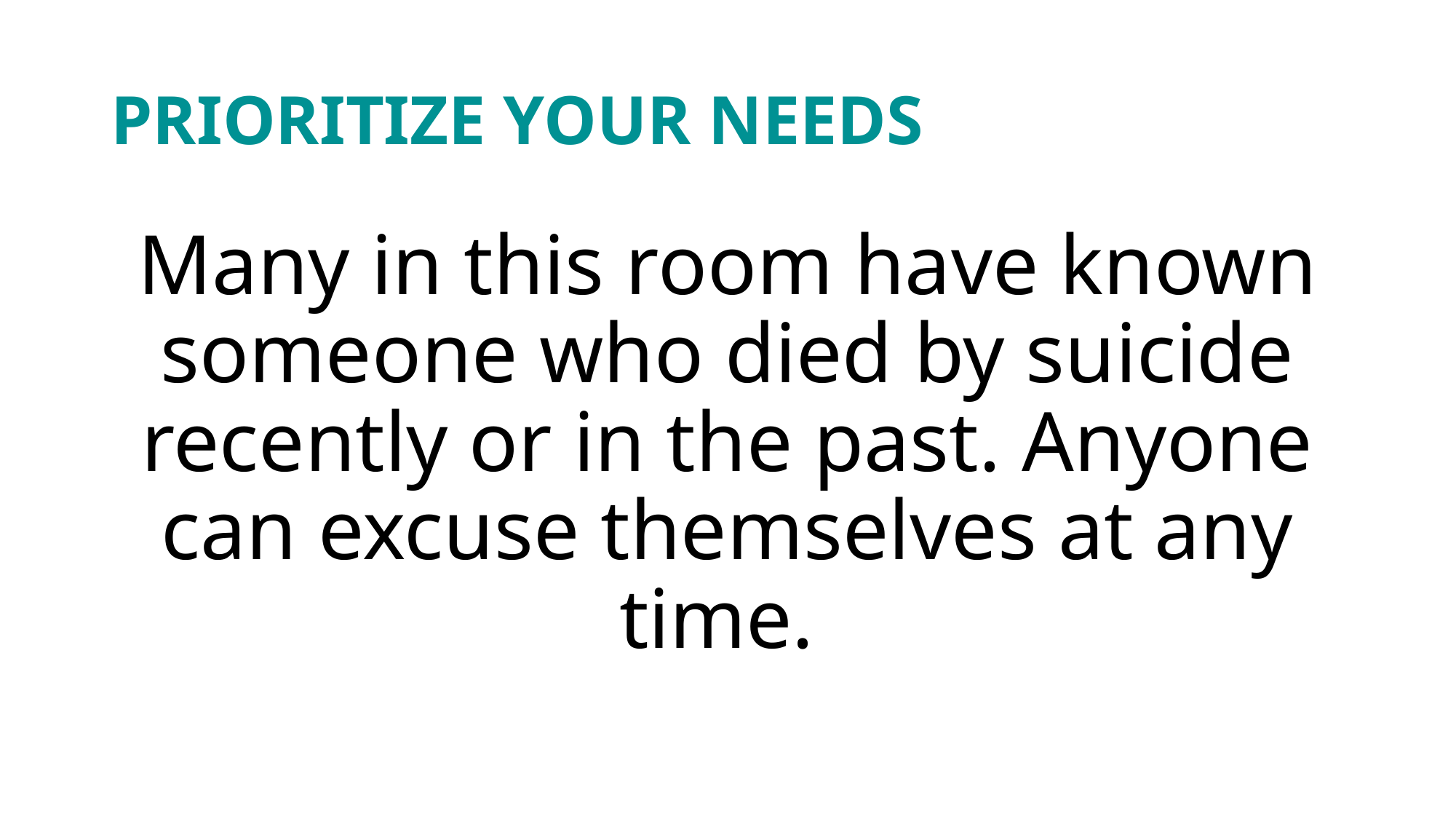

# PRIORITIZE YOUR NEEDS
Many in this room have known someone who died by suicide recently or in the past. Anyone can excuse themselves at any time.

## Slide 4
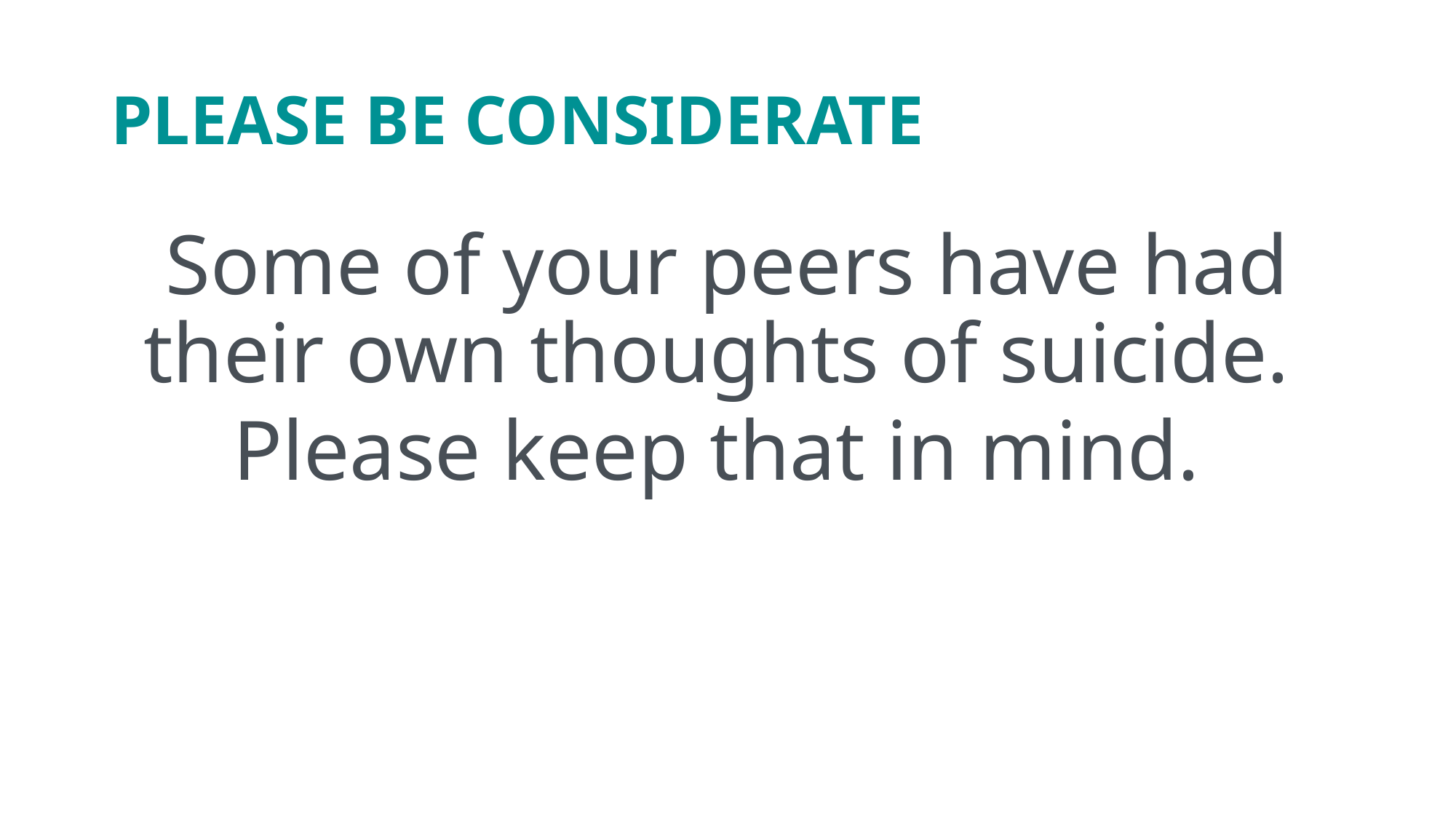

# PLEASE BE CONSIDERATE
Some of your peers have had their own thoughts of suicide.
Please keep that in mind.

## Slide 5
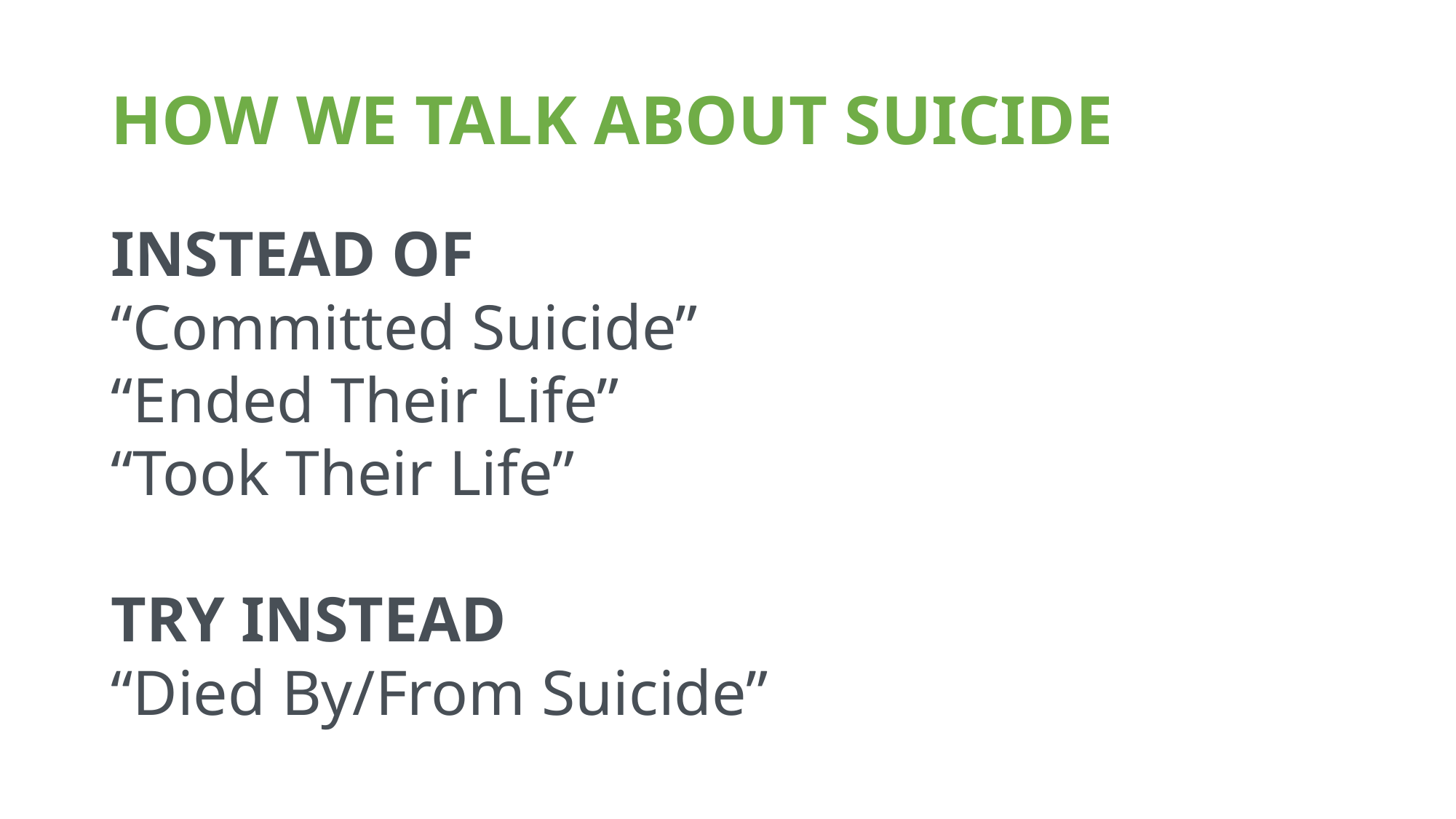

# HOW WE TALK ABOUT SUICIDE
INSTEAD OF
“Committed Suicide”
“Ended Their Life”
“Took Their Life”
TRY INSTEAD
“Died By/From Suicide”

## Slide 6
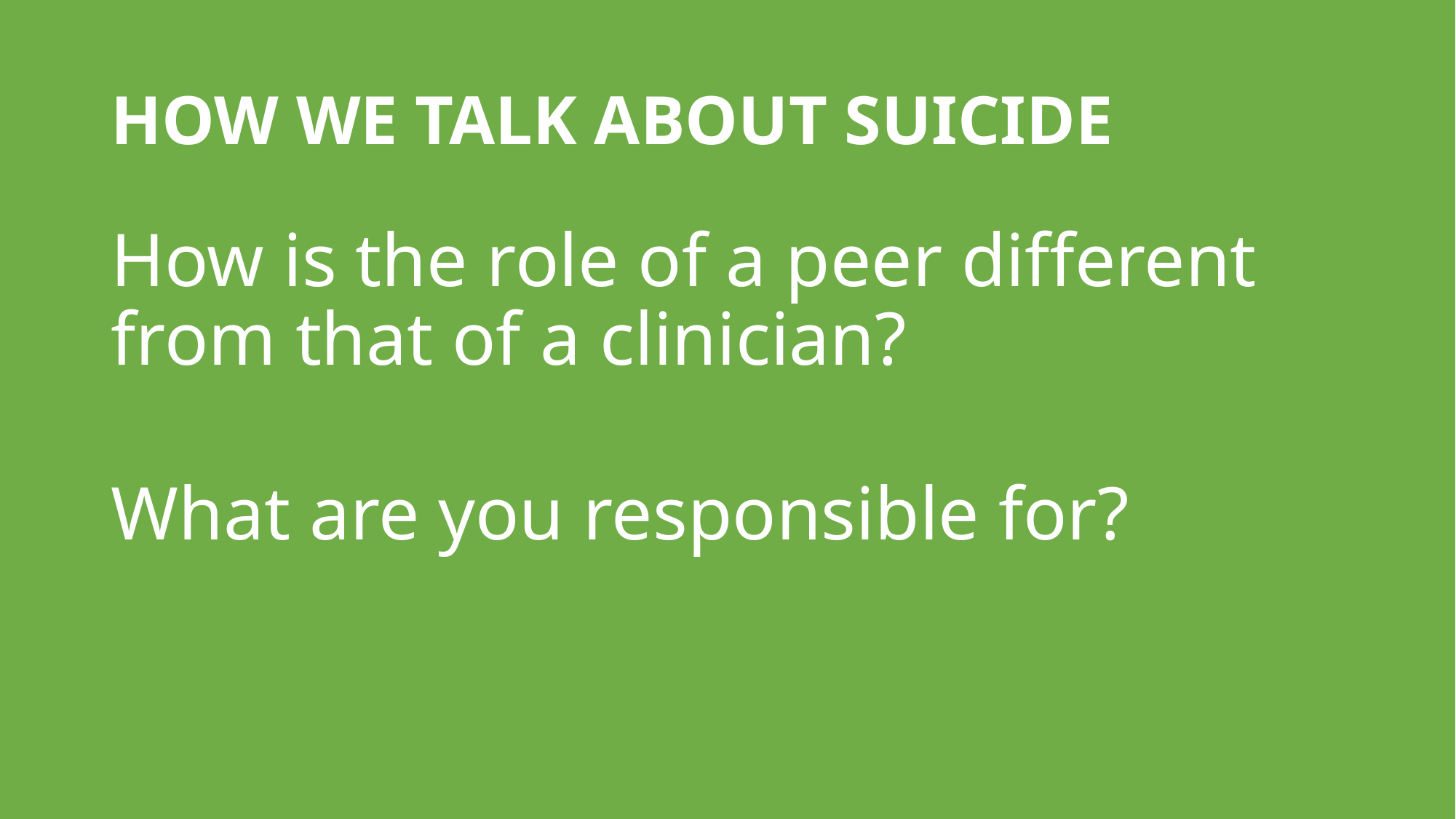

# HOW WE TALK ABOUT SUICIDE
How is the role of a peer different from that of a clinician?
What are you responsible for?

## Slide 7
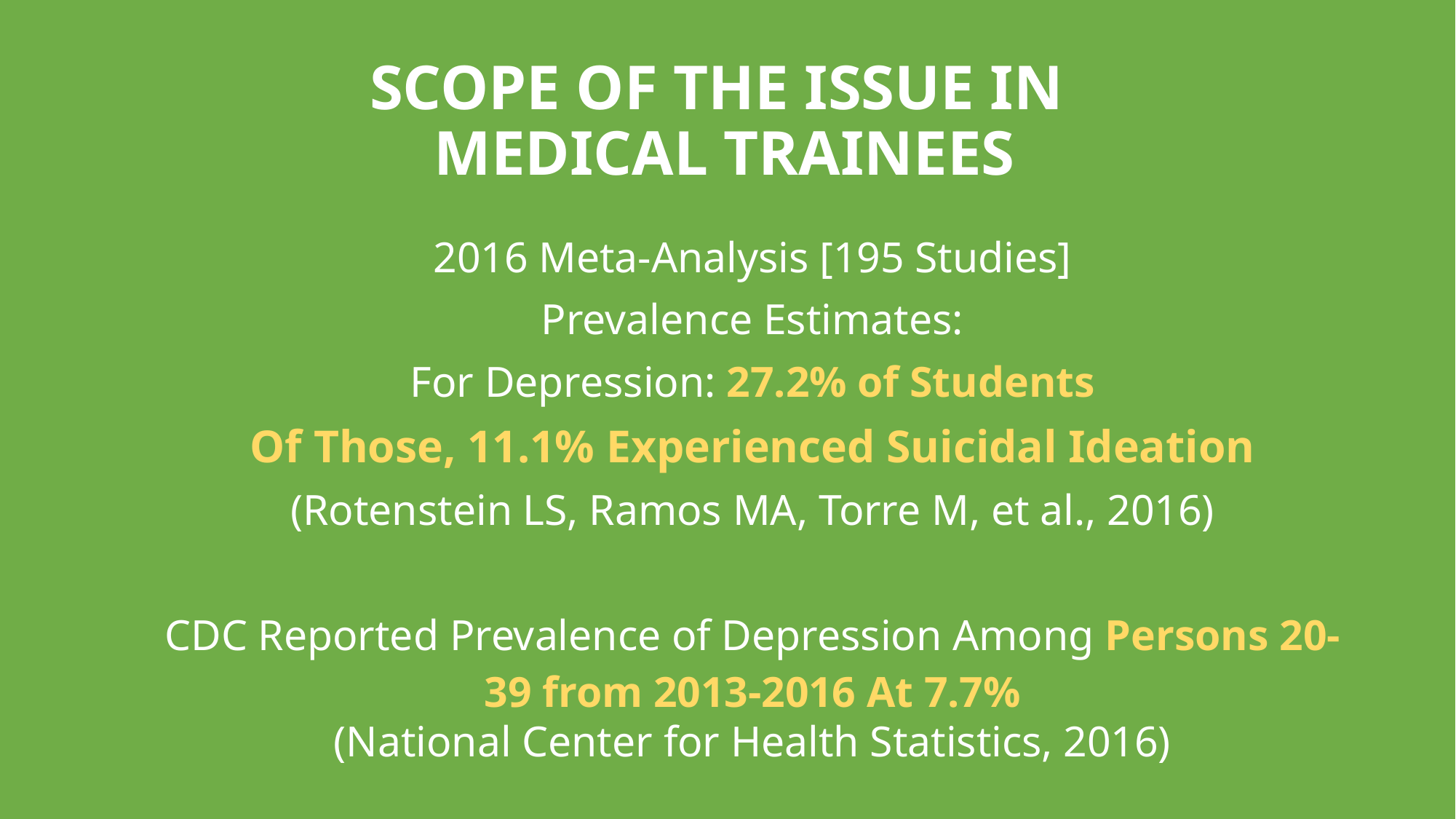

# SCOPE OF THE ISSUE IN MEDICAL TRAINEES
2016 Meta-Analysis [195 Studies]
Prevalence Estimates:
For Depression: 27.2% of Students
Of Those, 11.1% Experienced Suicidal Ideation
(Rotenstein LS, Ramos MA, Torre M, et al., 2016)
CDC Reported Prevalence of Depression Among Persons 20-39 from 2013-2016 At 7.7%
(National Center for Health Statistics, 2016)

## Slide 8
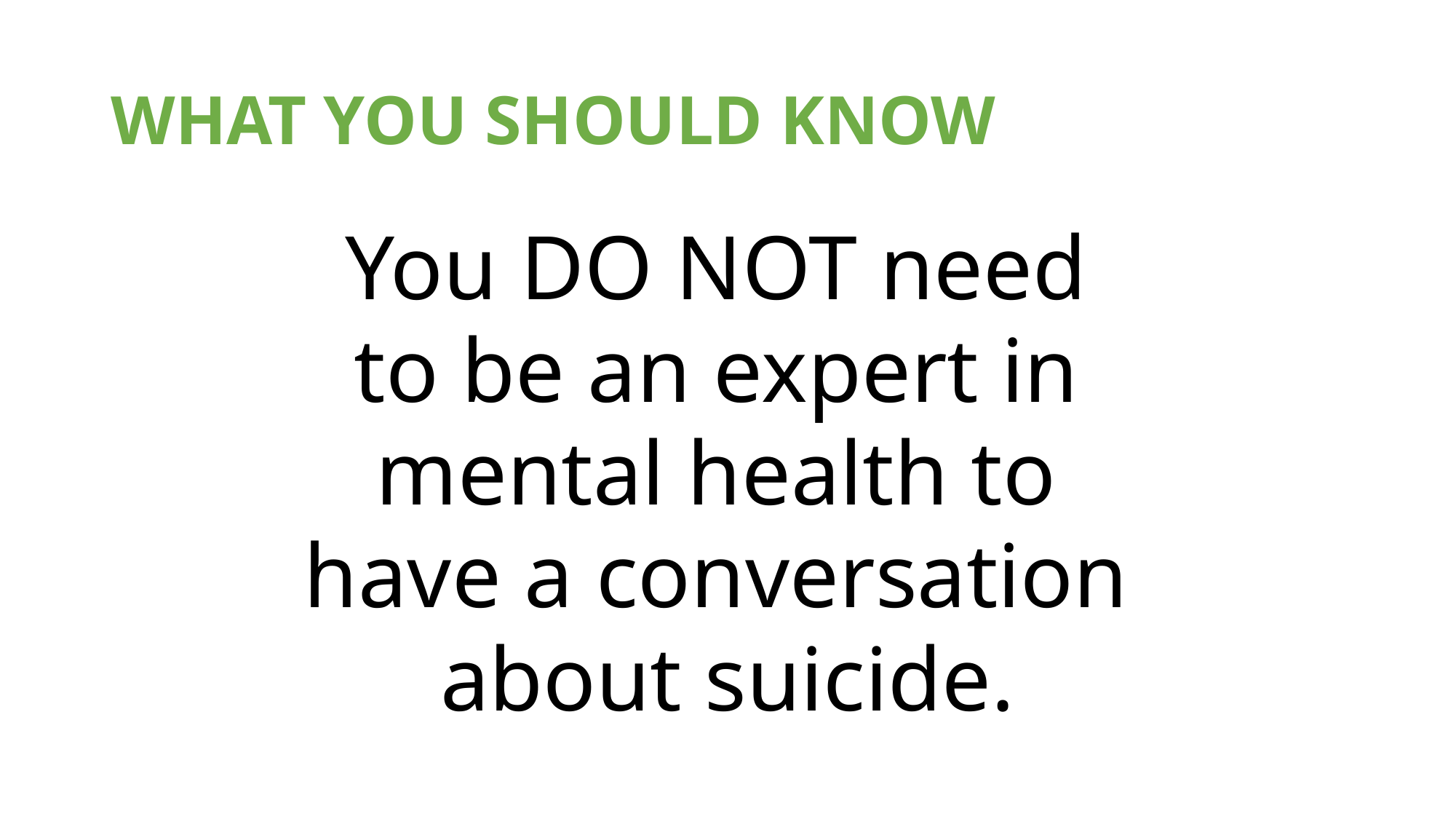

# WHAT YOU SHOULD KNOW
You DO NOT need
to be an expert in
mental health to
have a conversation
about suicide.

## Slide 9
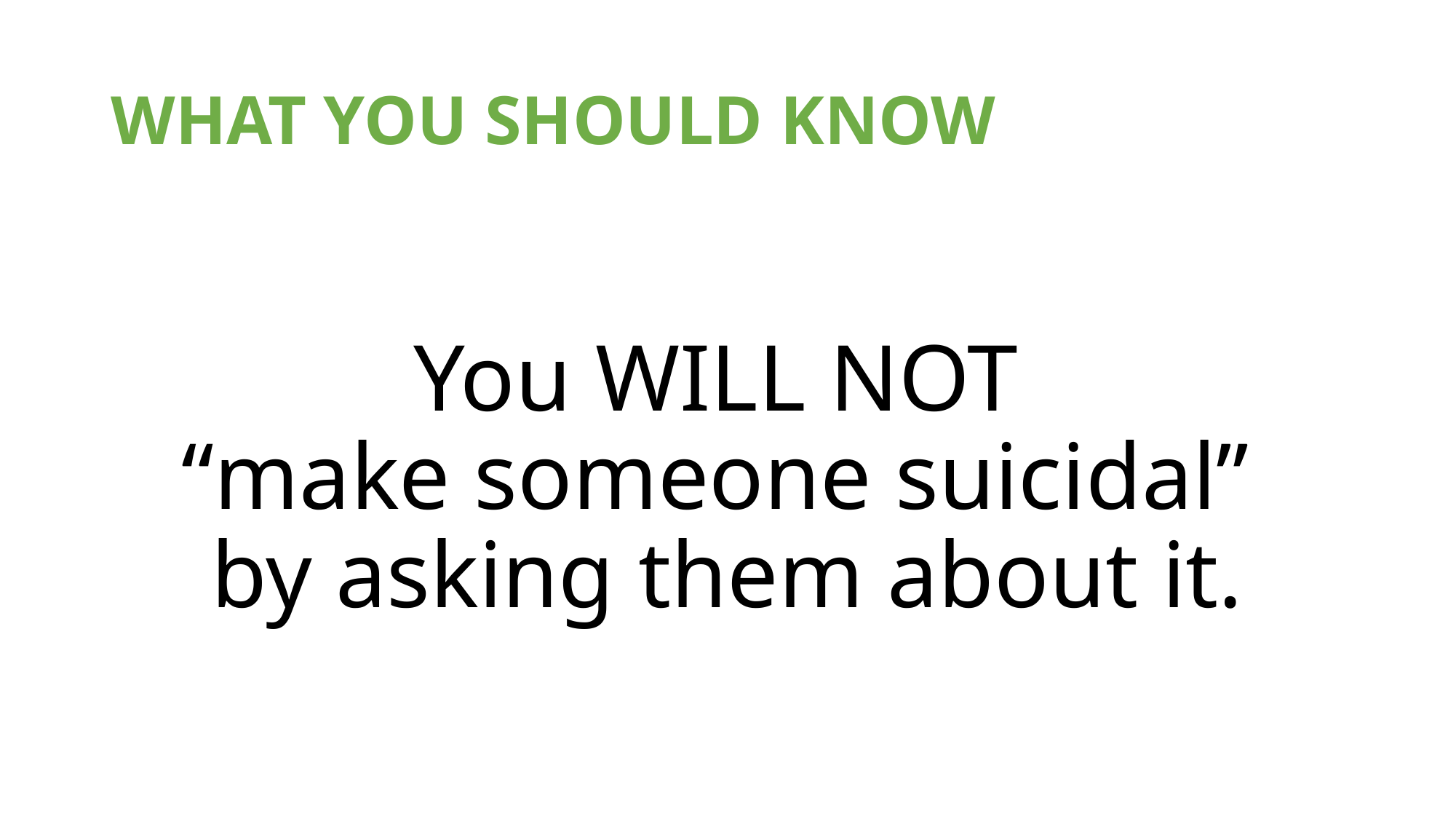

# WHAT YOU SHOULD KNOW
You WILL NOT
“make someone suicidal”
by asking them about it.

## Slide 10
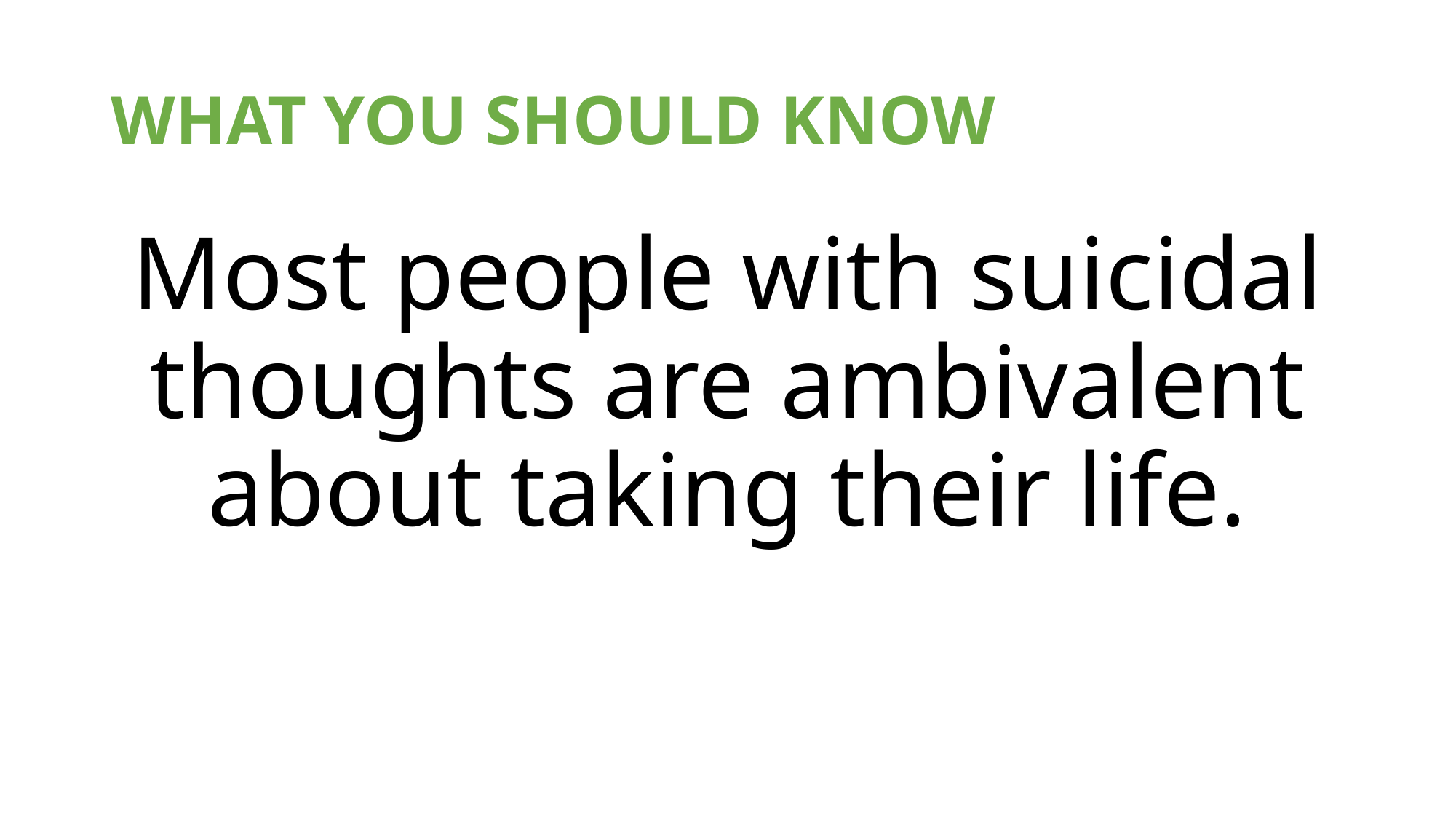

# WHAT YOU SHOULD KNOW
Most people with suicidal thoughts are ambivalent about taking their life.

## Slide 11
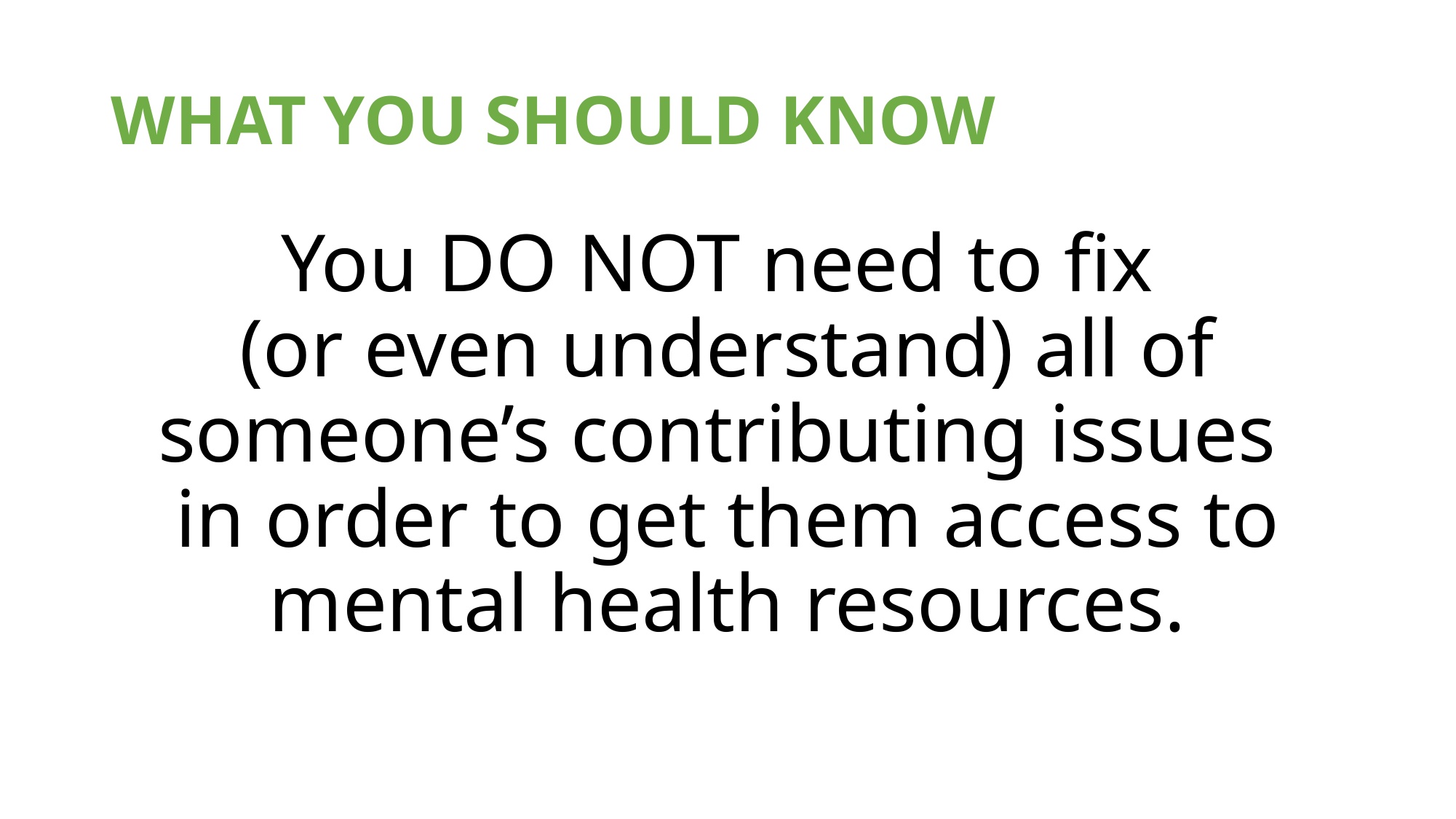

# WHAT YOU SHOULD KNOW
You DO NOT need to fix
(or even understand) all of someone’s contributing issues
in order to get them access to mental health resources.

## Slide 12
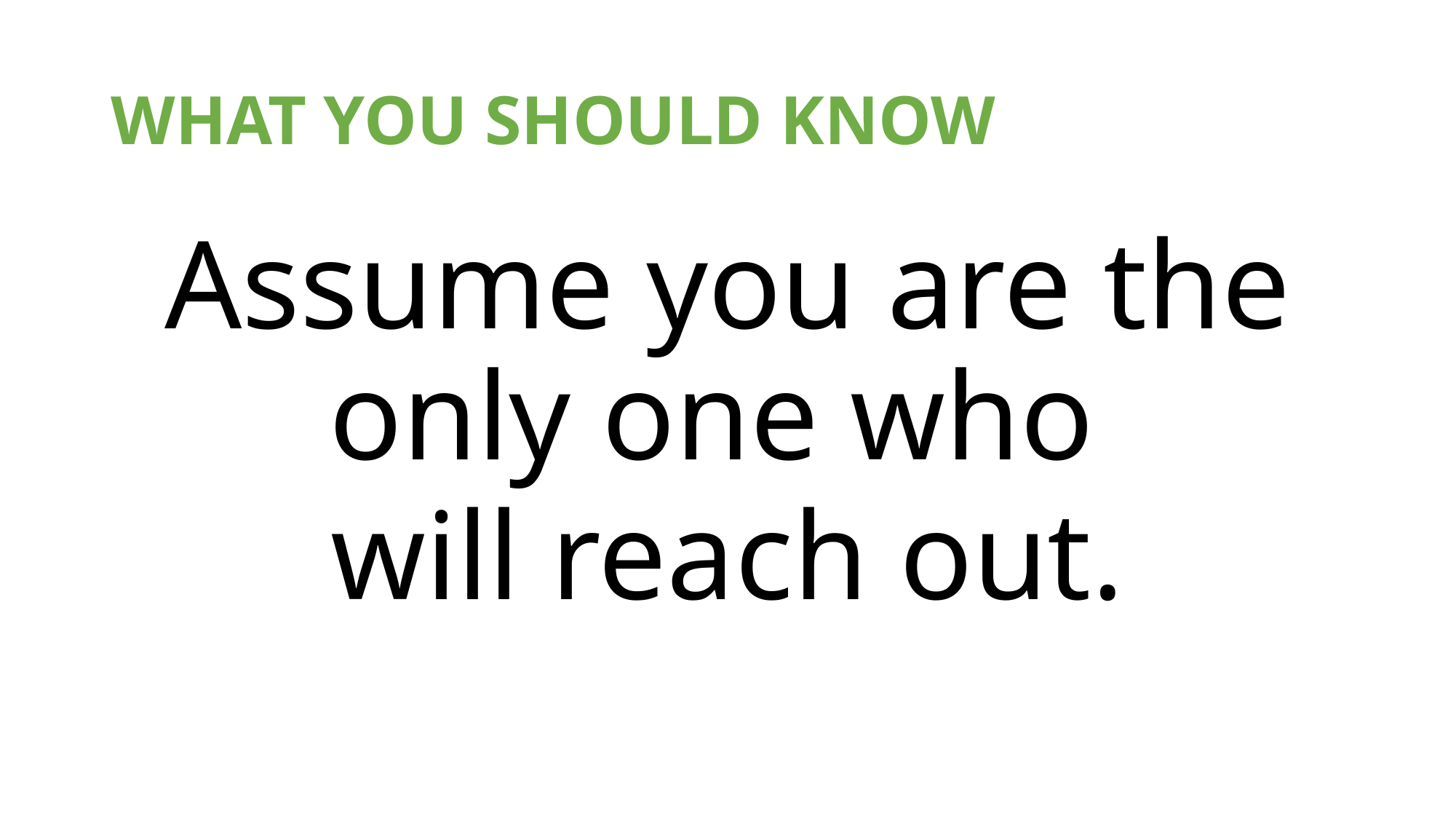

# WHAT YOU SHOULD KNOW
Assume you are the only one who
will reach out.

## Slide 13
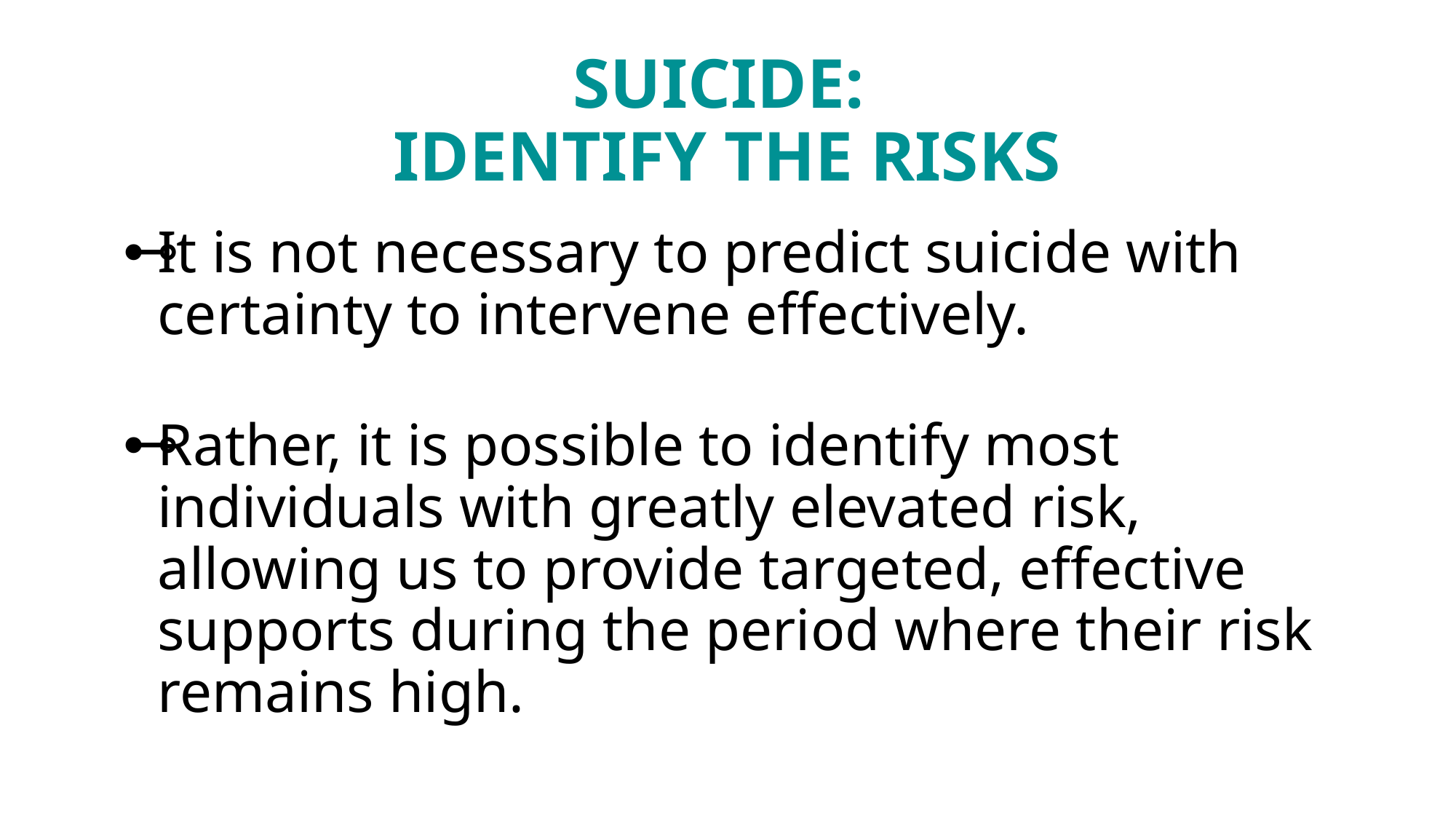

# SUICIDE: IDENTIFY THE RISKS
It is not necessary to predict suicide with certainty to intervene effectively.
Rather, it is possible to identify most individuals with greatly elevated risk, allowing us to provide targeted, effective supports during the period where their risk remains high.

## Slide 14
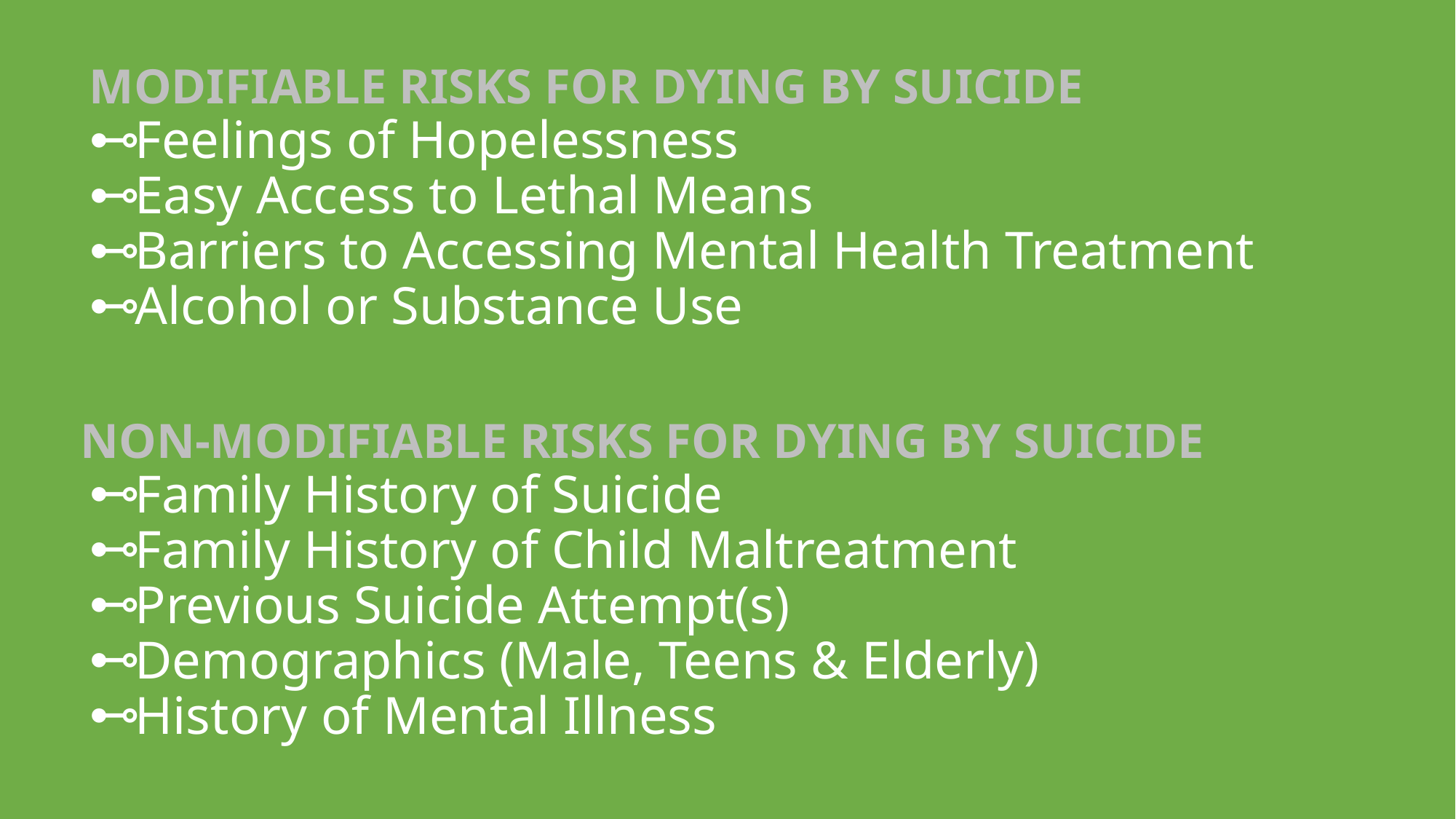

MODIFIABLE RISKS FOR DYING BY SUICIDE
Feelings of Hopelessness
Easy Access to Lethal Means
Barriers to Accessing Mental Health Treatment
Alcohol or Substance Use
NON-MODIFIABLE RISKS FOR DYING BY SUICIDE
Family History of Suicide
Family History of Child Maltreatment
Previous Suicide Attempt(s)
Demographics (Male, Teens & Elderly)
History of Mental Illness

## Slide 15
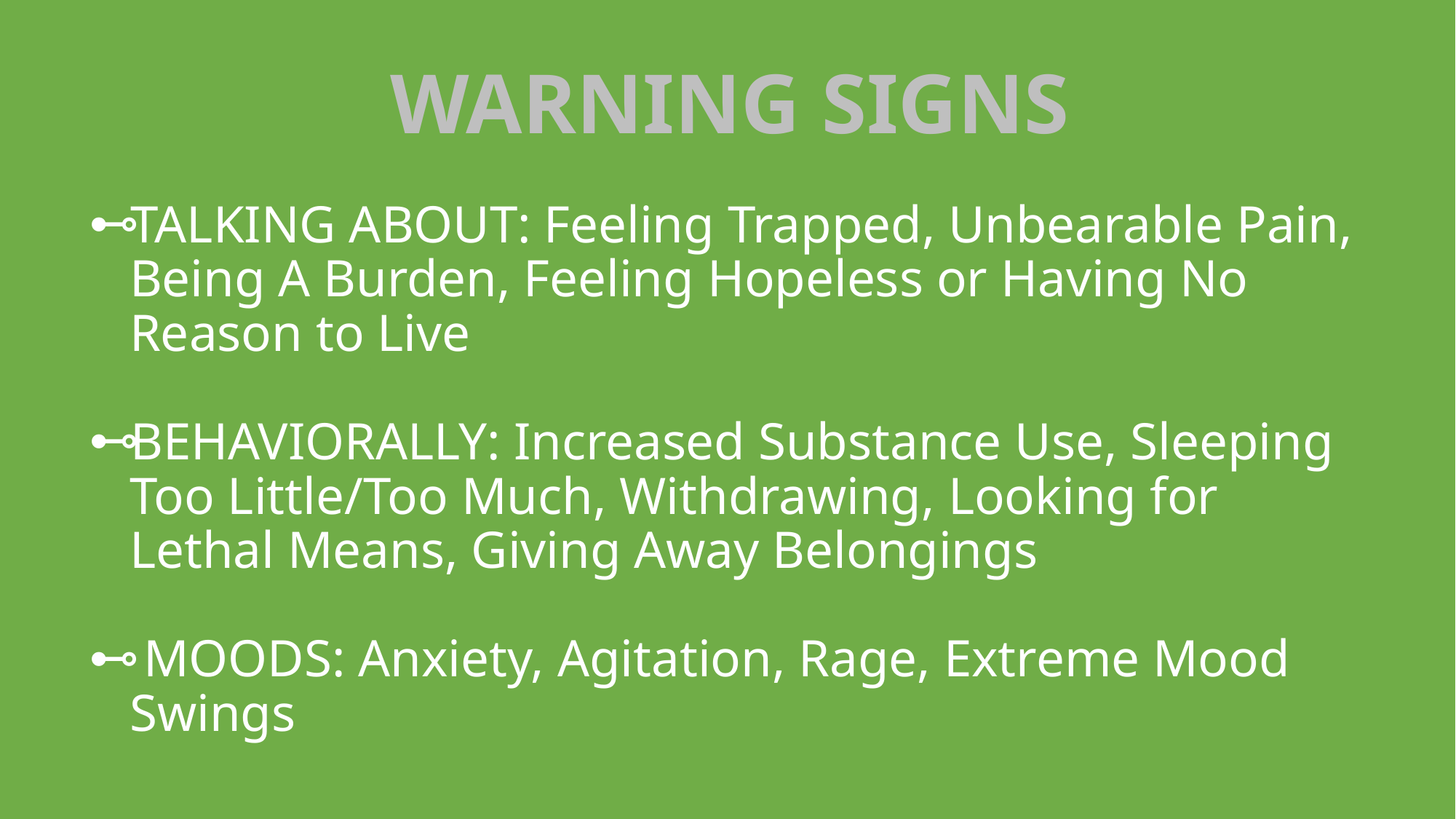

WARNING SIGNS
TALKING ABOUT: Feeling Trapped, Unbearable Pain, Being A Burden, Feeling Hopeless or Having No Reason to Live
BEHAVIORALLY: Increased Substance Use, Sleeping Too Little/Too Much, Withdrawing, Looking for Lethal Means, Giving Away Belongings
 MOODS: Anxiety, Agitation, Rage, Extreme Mood Swings

## Slide 16
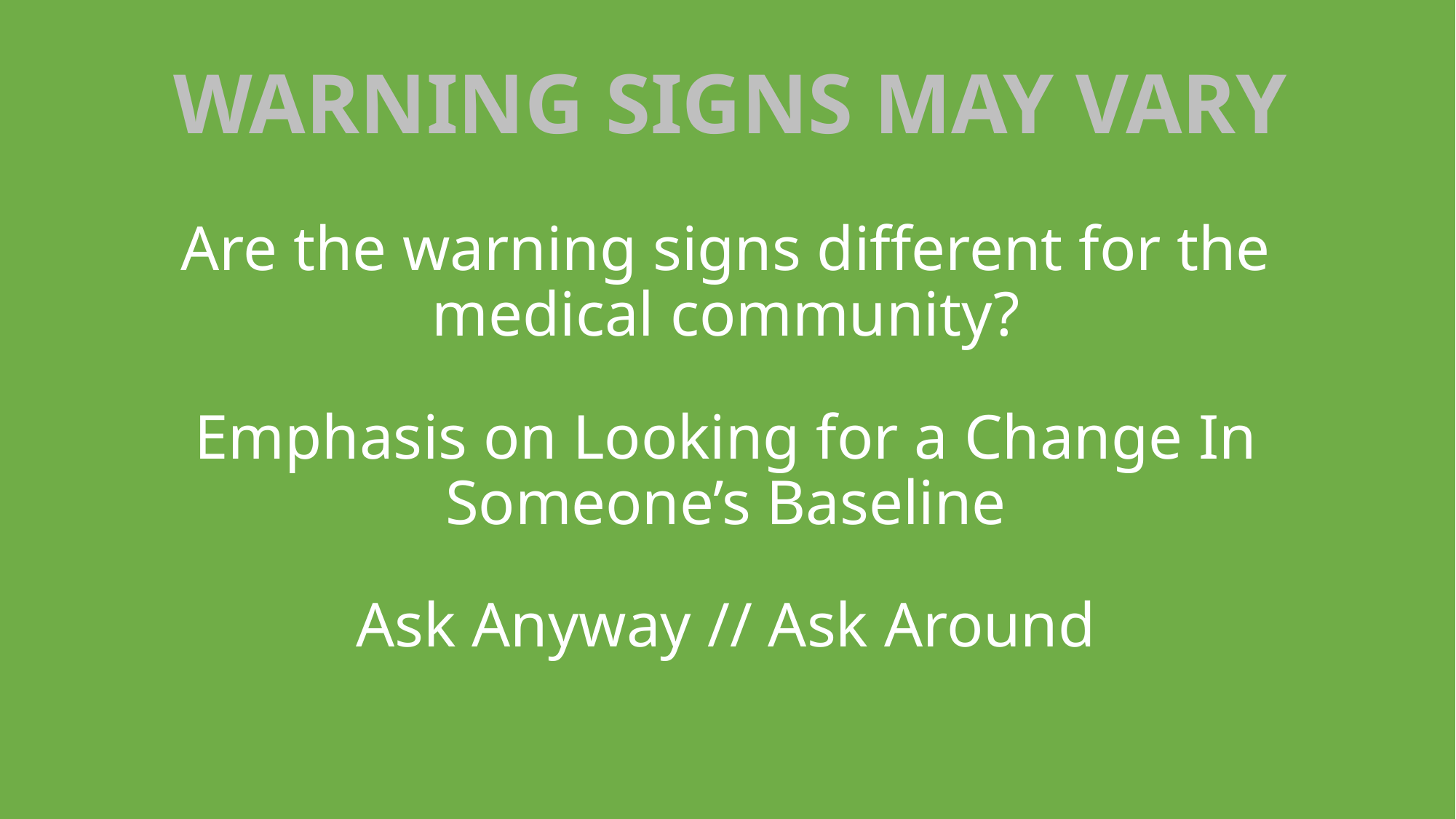

WARNING SIGNS MAY VARY
Are the warning signs different for the medical community?
Emphasis on Looking for a Change In Someone’s Baseline
Ask Anyway // Ask Around

## Slide 17
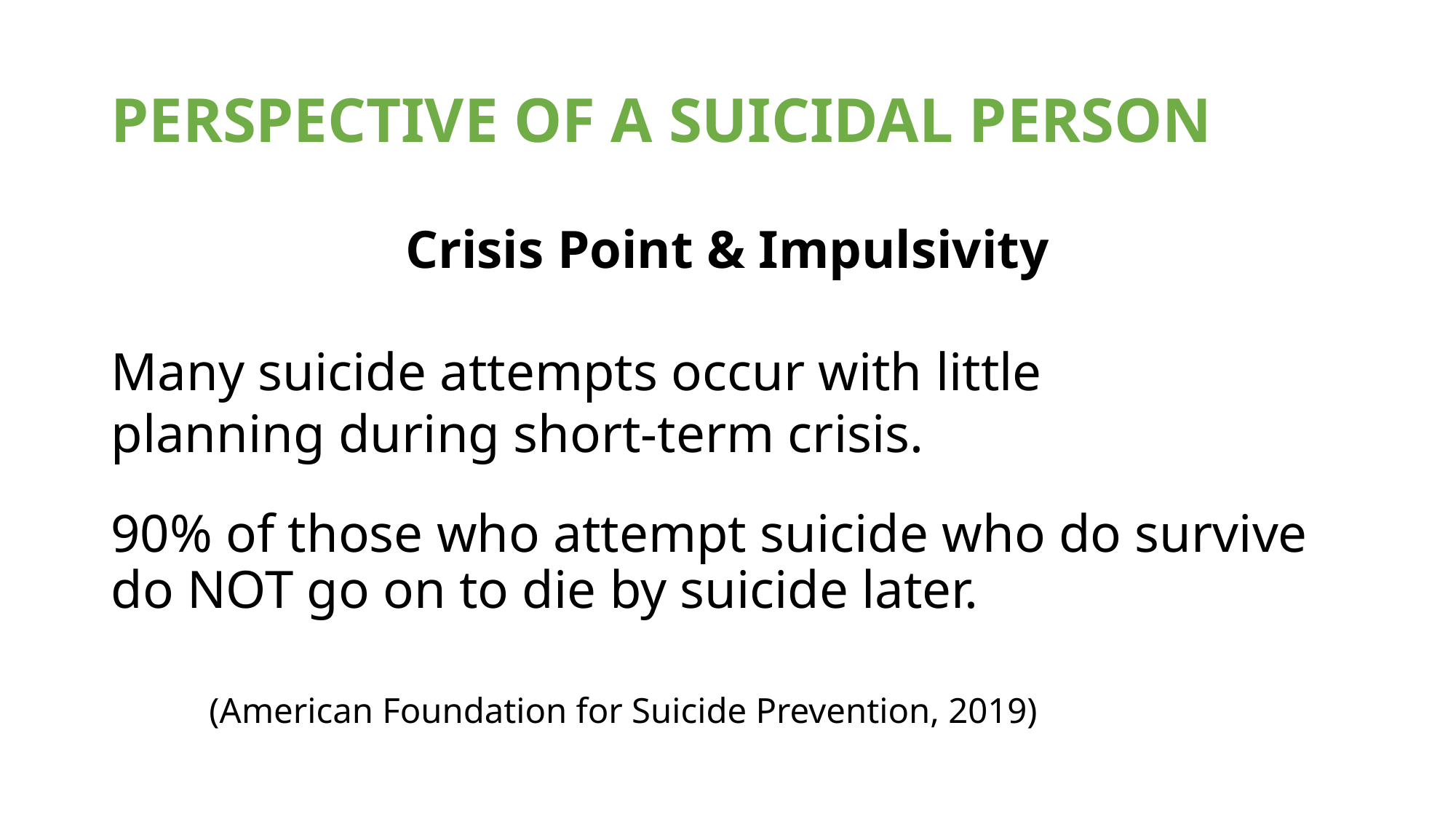

# PERSPECTIVE OF A SUICIDAL PERSON
Crisis Point & Impulsivity
Many suicide attempts occur with little
planning during short-term crisis.
90% of those who attempt suicide who do survive do NOT go on to die by suicide later.
(American Foundation for Suicide Prevention, 2019)

## Slide 18
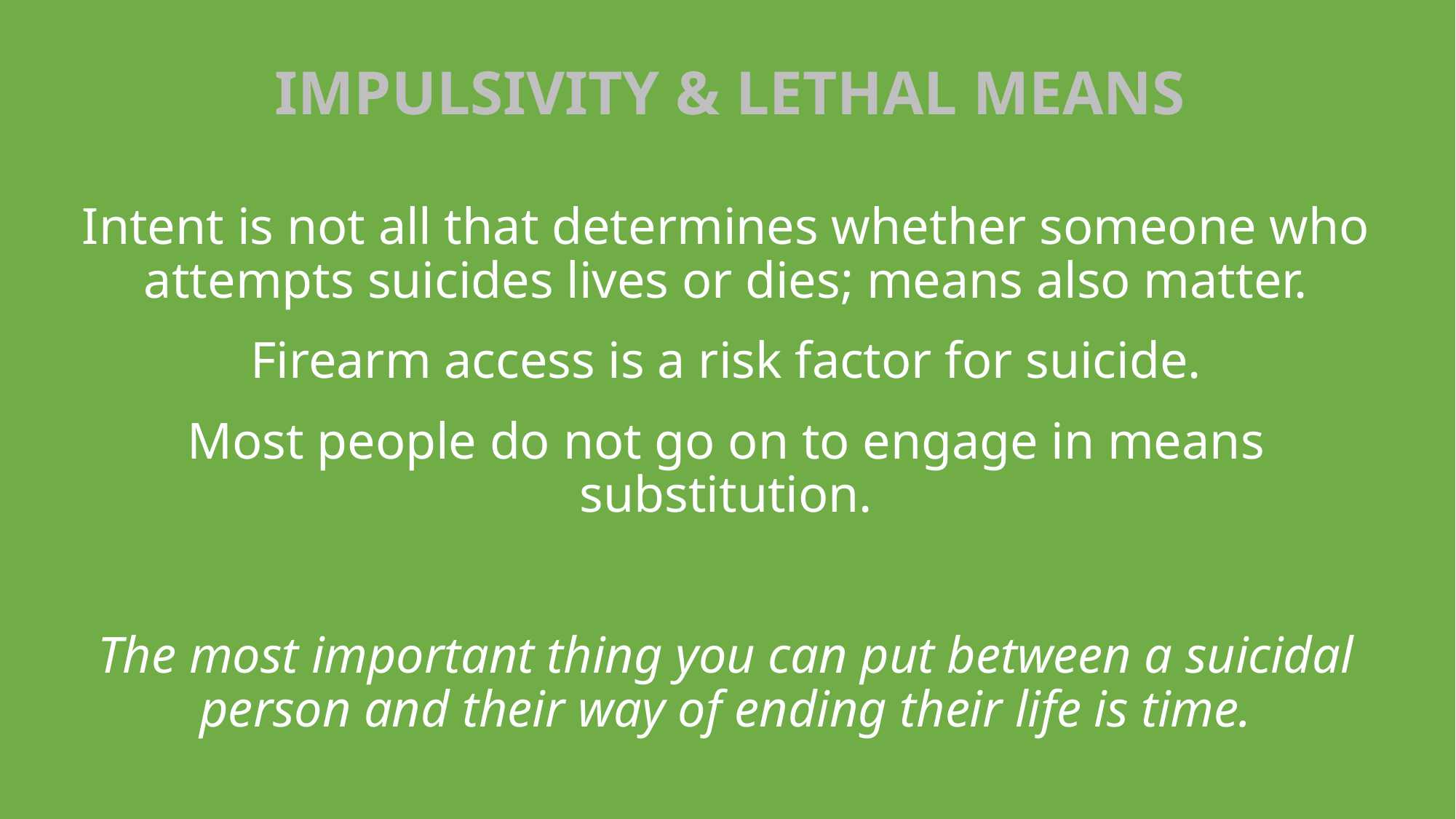

IMPULSIVITY & LETHAL MEANS
Intent is not all that determines whether someone who attempts suicides lives or dies; means also matter.
Firearm access is a risk factor for suicide.
Most people do not go on to engage in means substitution.
The most important thing you can put between a suicidal person and their way of ending their life is time.

## Slide 19
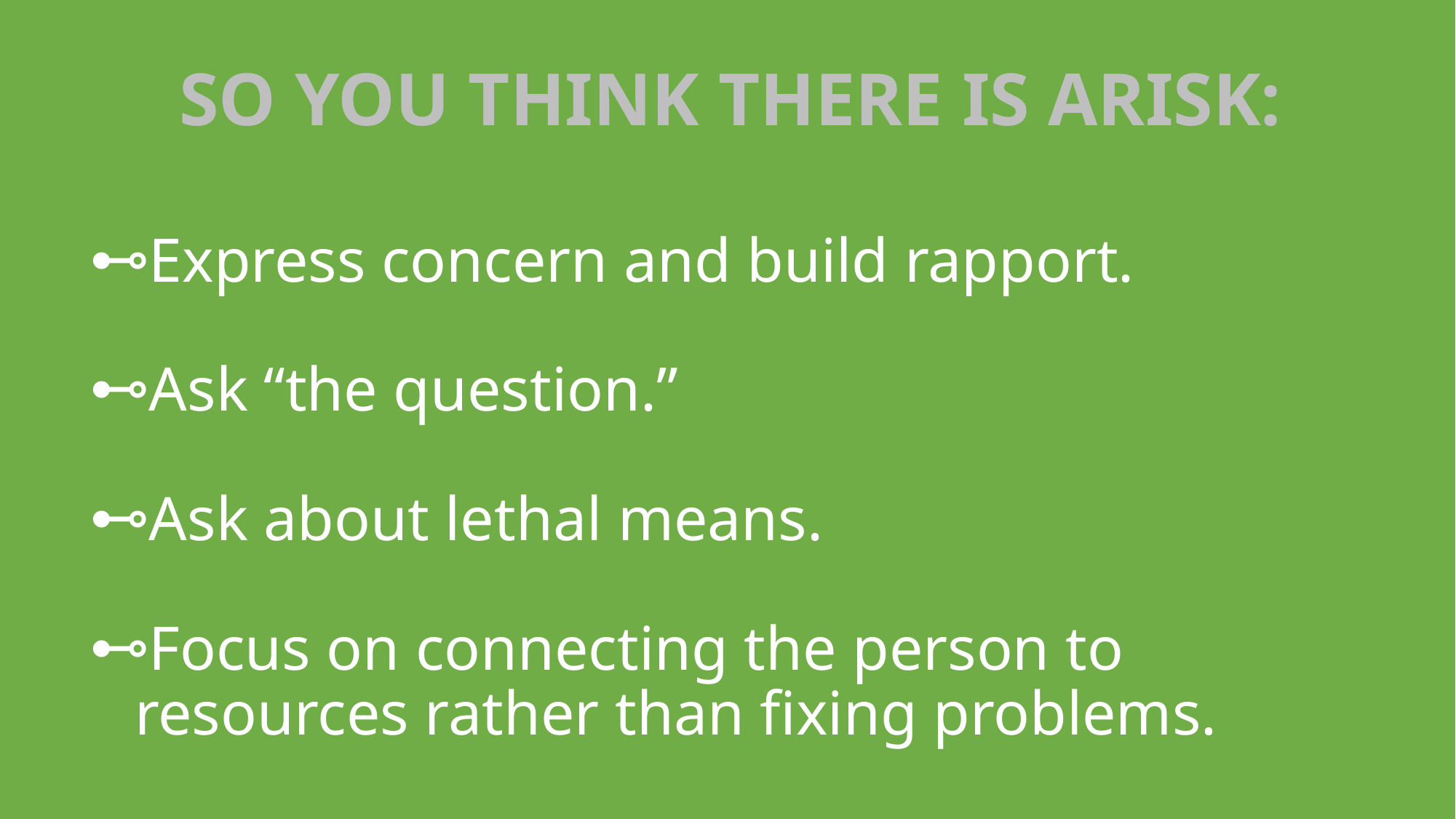

SO YOU THINK THERE IS ARISK:
Express concern and build rapport.
Ask “the question.”
Ask about lethal means.
Focus on connecting the person to resources rather than fixing problems.

## Slide 20
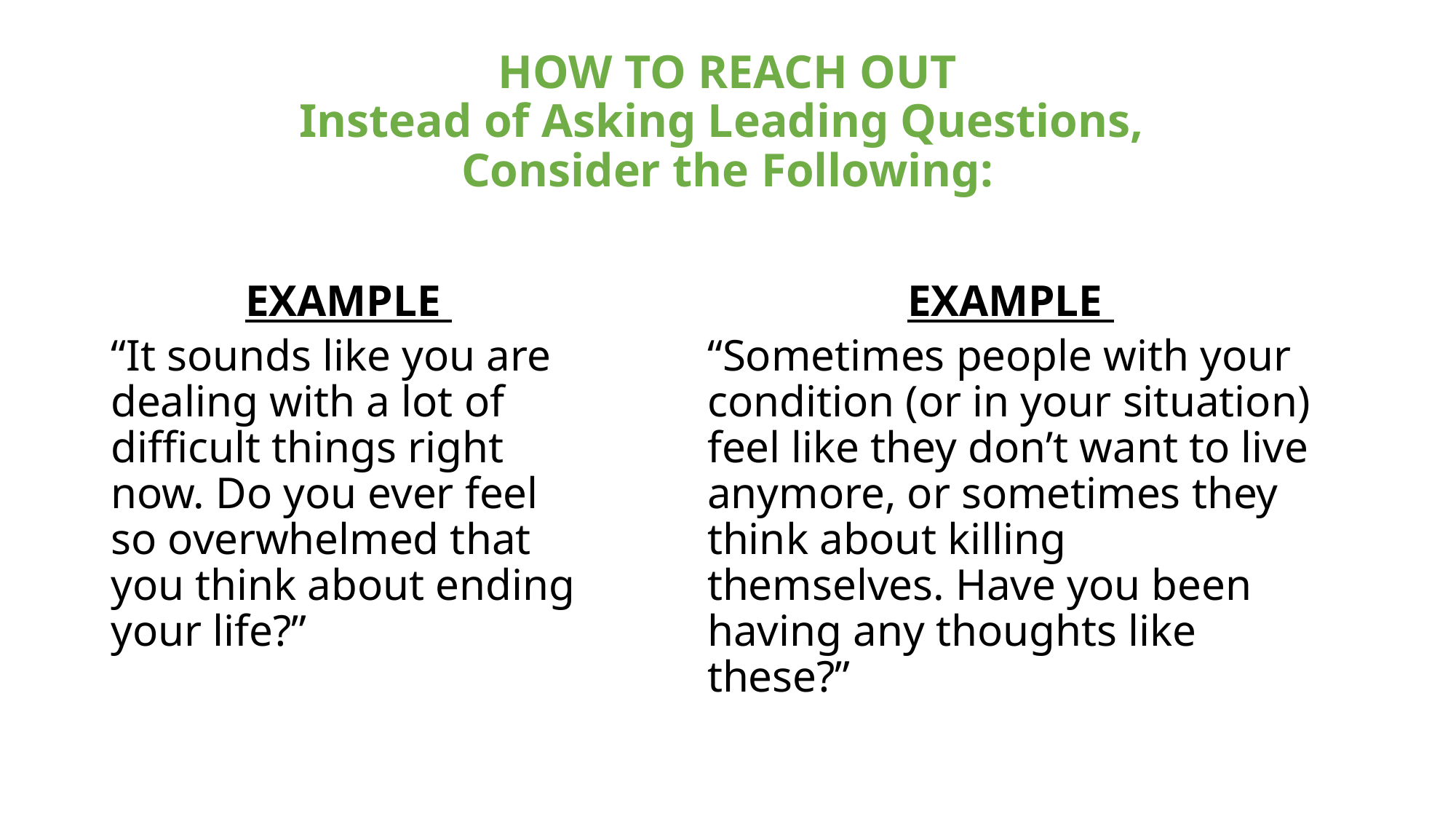

# HOW TO REACH OUT Instead of Asking Leading Questions, Consider the Following:
EXAMPLE
“It sounds like you are dealing with a lot of difficult things right now. Do you ever feel so overwhelmed that you think about ending your life?”
EXAMPLE
“Sometimes people with your condition (or in your situation) feel like they don’t want to live anymore, or sometimes they think about killing themselves. Have you been having any thoughts like these?”

## Slide 21
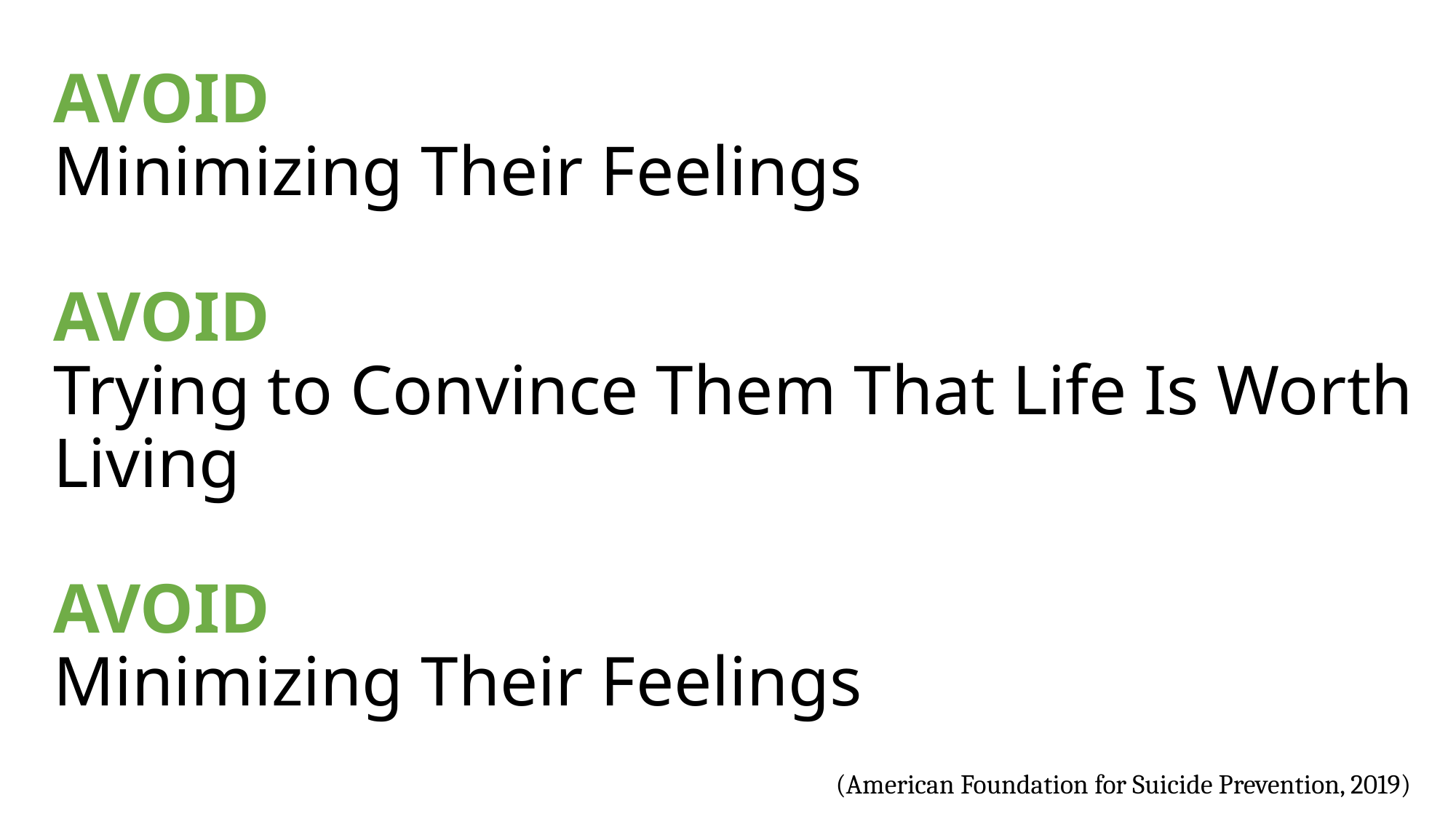

# AVOID Minimizing Their Feelings AVOID Trying to Convince Them That Life Is Worth Living AVOID Minimizing Their Feelings
(American Foundation for Suicide Prevention, 2019)

## Slide 22
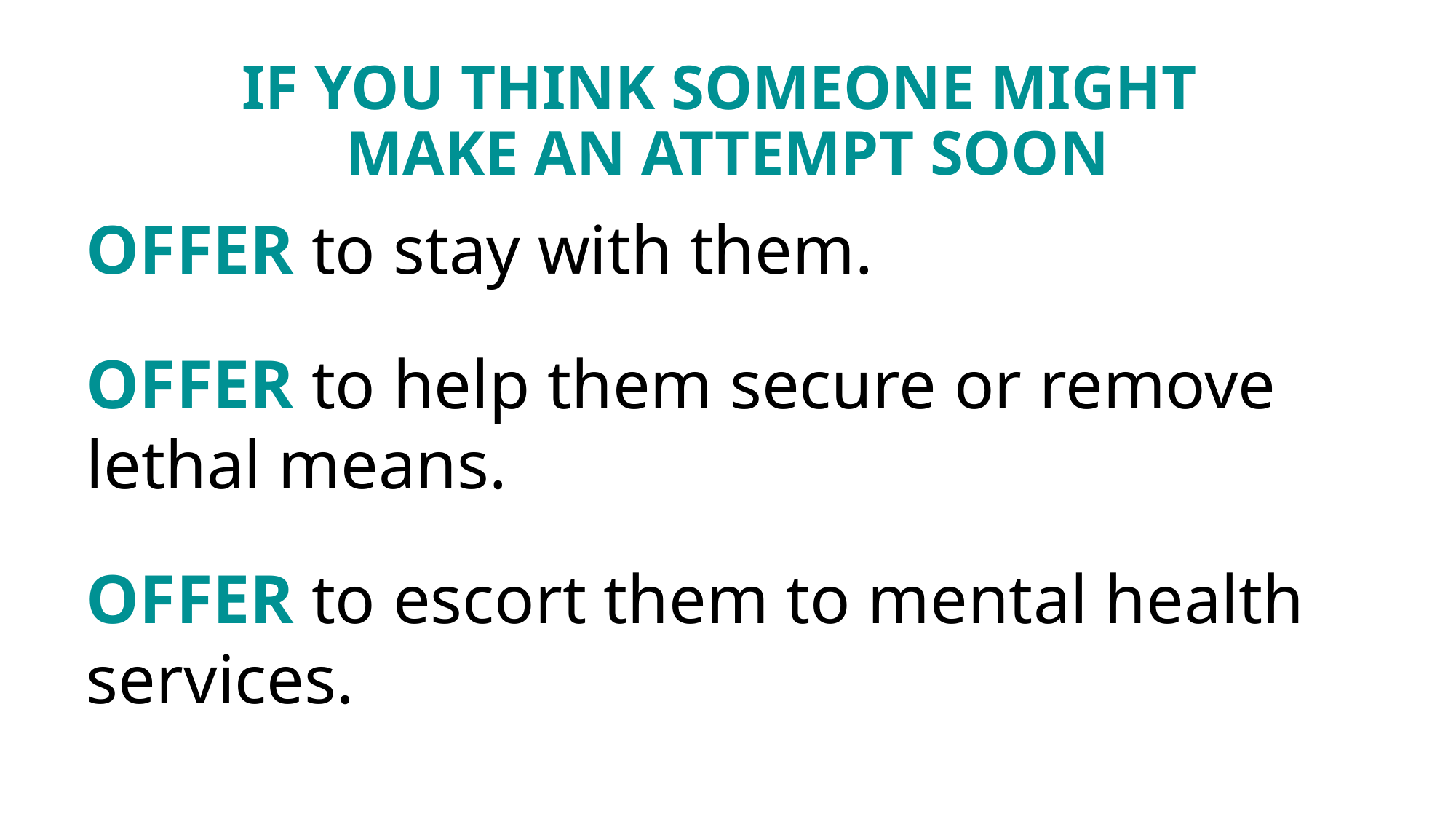

# IF YOU THINK SOMEONE MIGHT MAKE AN ATTEMPT SOON
OFFER to stay with them.
OFFER to help them secure or remove lethal means.
OFFER to escort them to mental health services.

## Slide 23
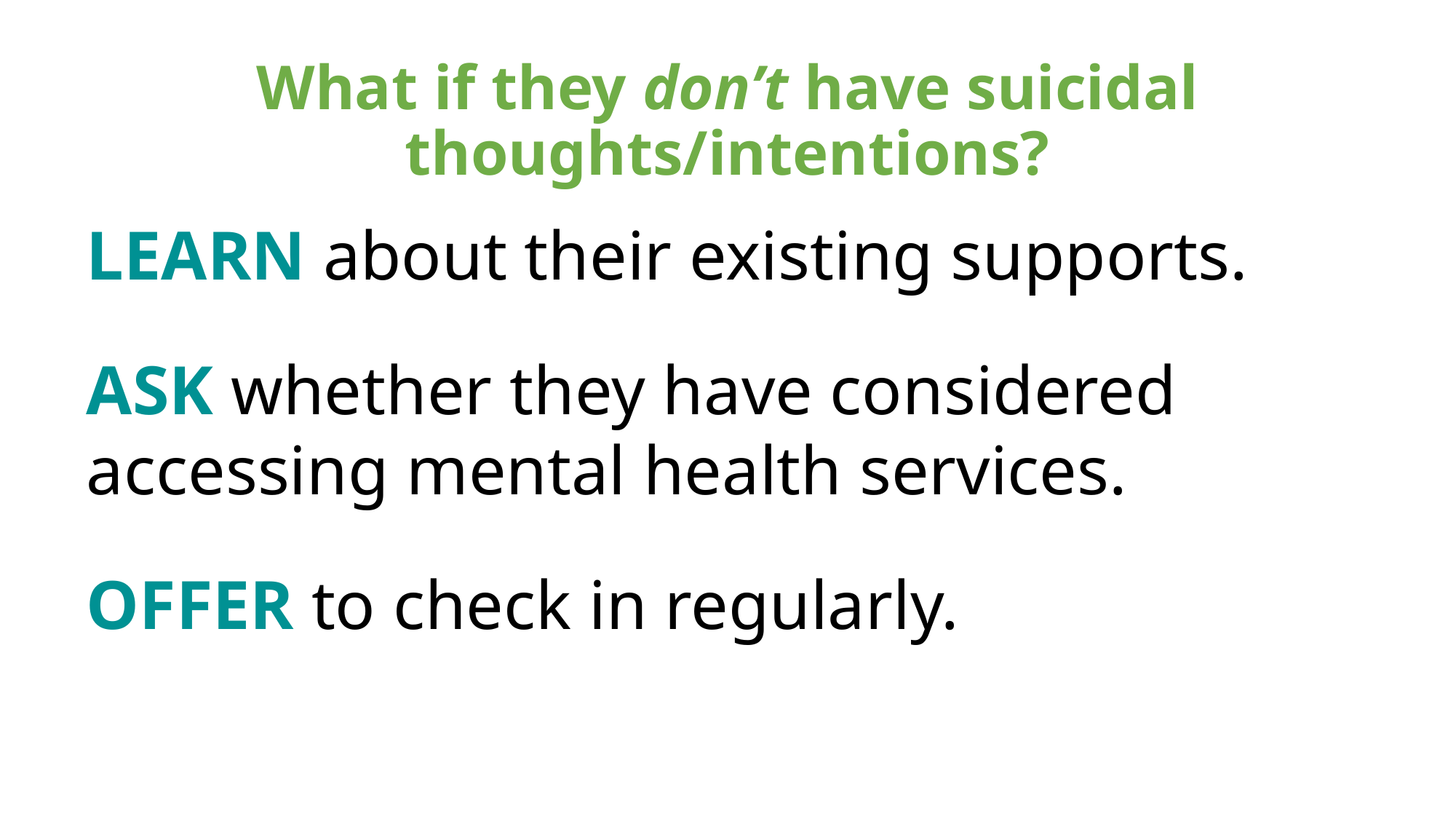

# What if they don’t have suicidal thoughts/intentions?
LEARN about their existing supports.
ASK whether they have considered accessing mental health services.
OFFER to check in regularly.

## Slide 24
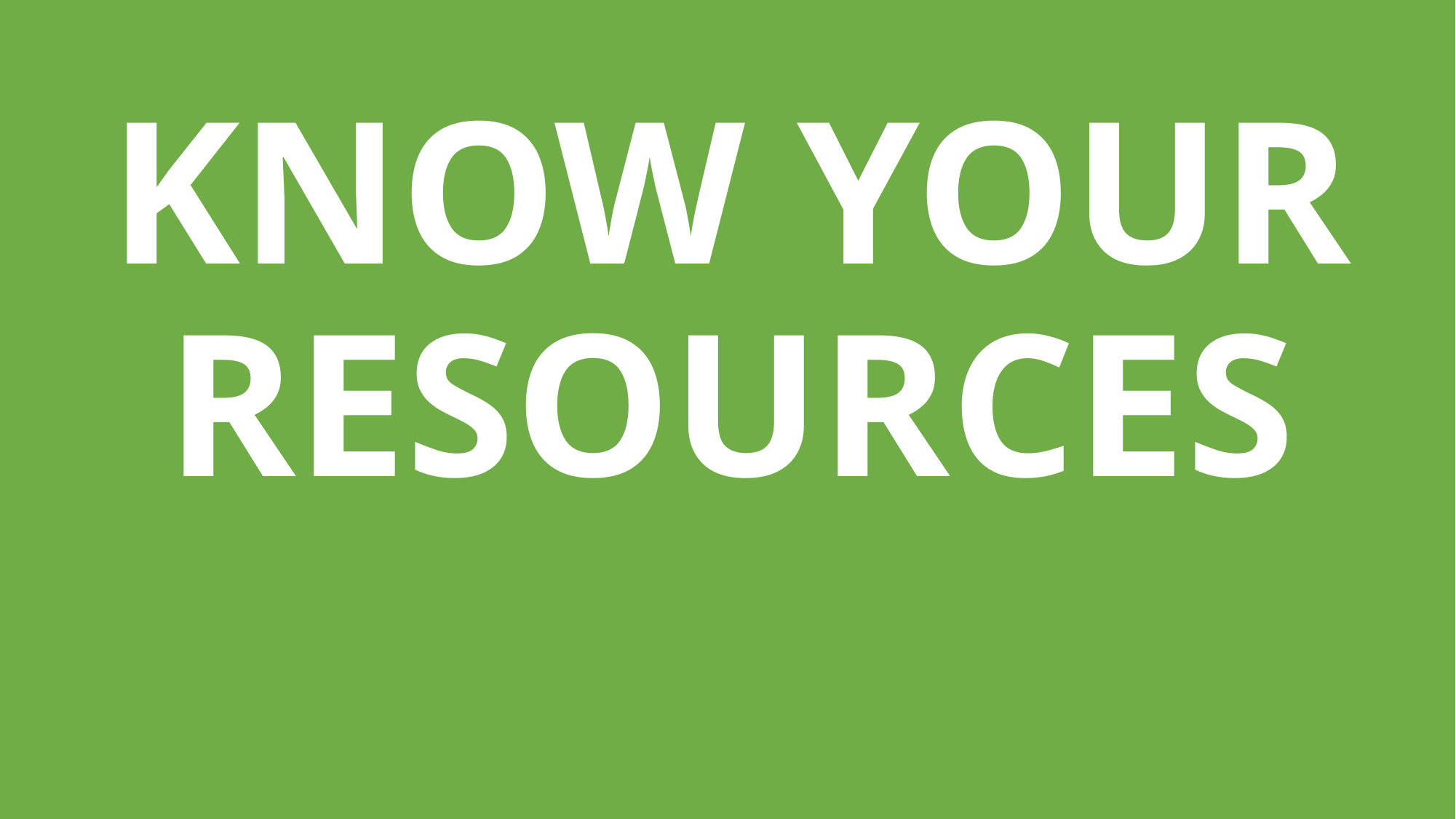

KNOW YOUR RESOURCES

## Slide 25
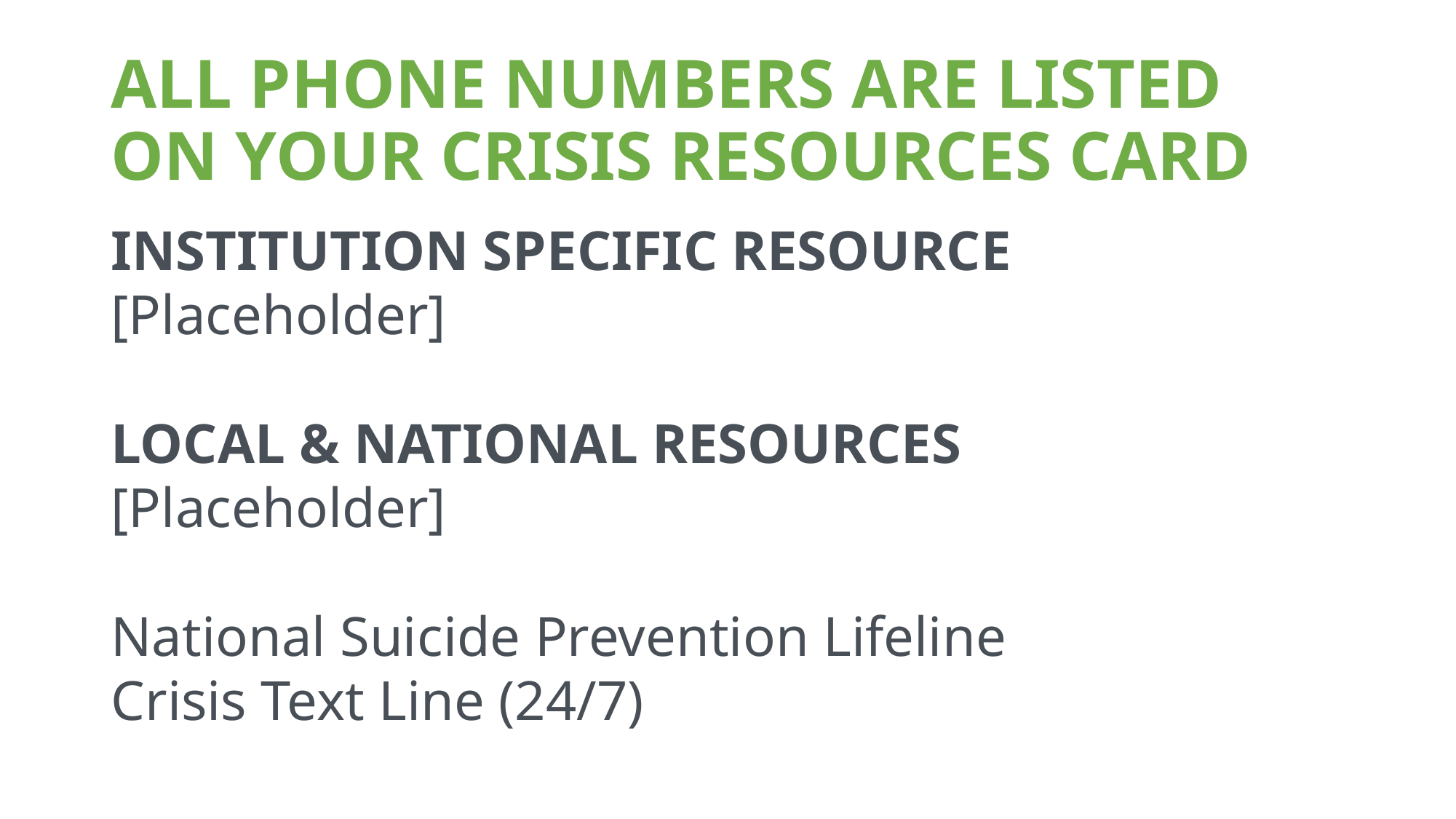

# ALL PHONE NUMBERS ARE LISTED ON YOUR CRISIS RESOURCES CARD
INSTITUTION SPECIFIC RESOURCE
[Placeholder]
LOCAL & NATIONAL RESOURCES
[Placeholder]
National Suicide Prevention Lifeline
Crisis Text Line (24/7)

## Slide 26
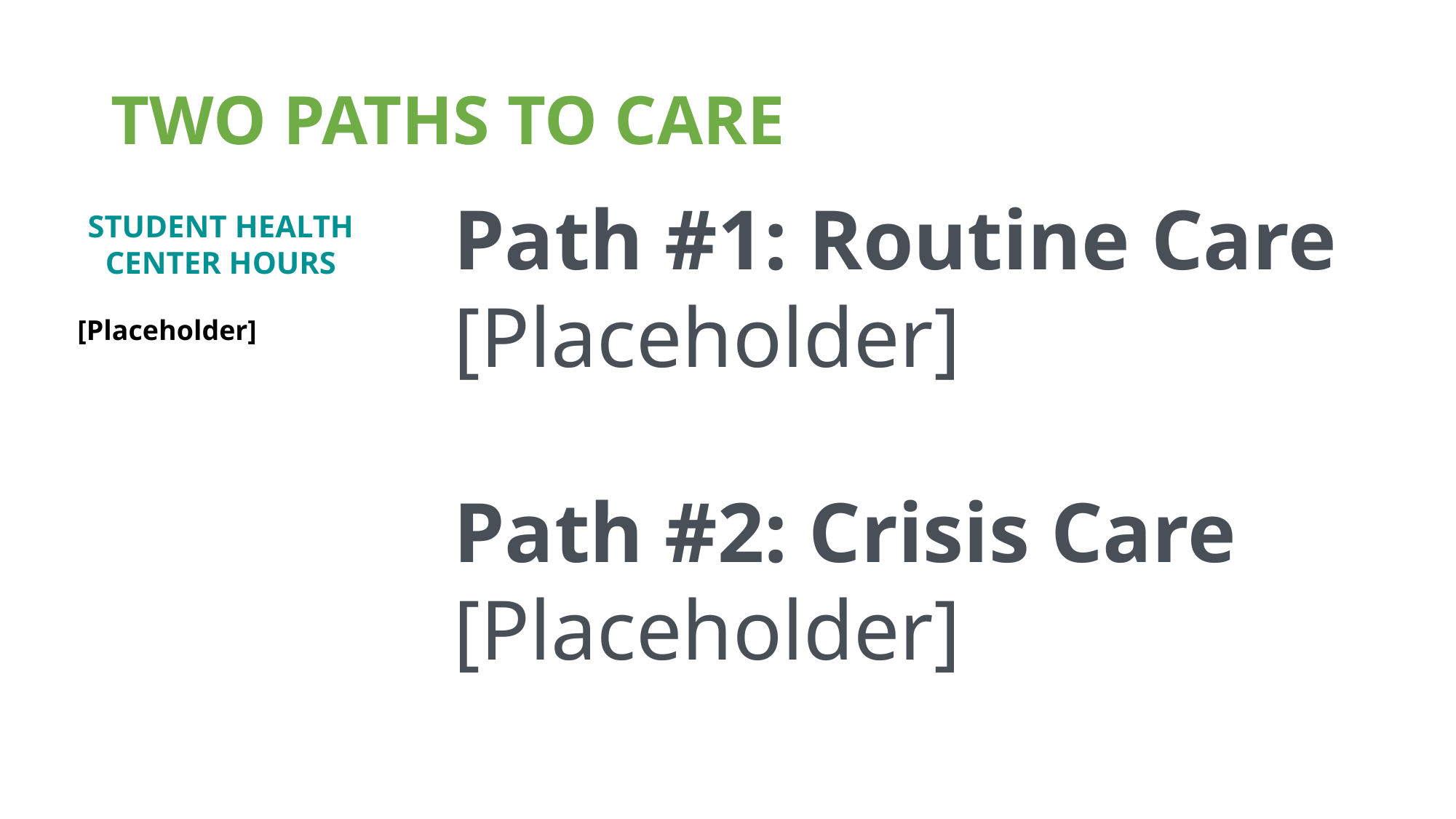

# TWO PATHS TO CARE
Path #1: Routine Care
[Placeholder]
Path #2: Crisis Care
[Placeholder]
STUDENT HEALTH CENTER HOURS
[Placeholder]

## Slide 27
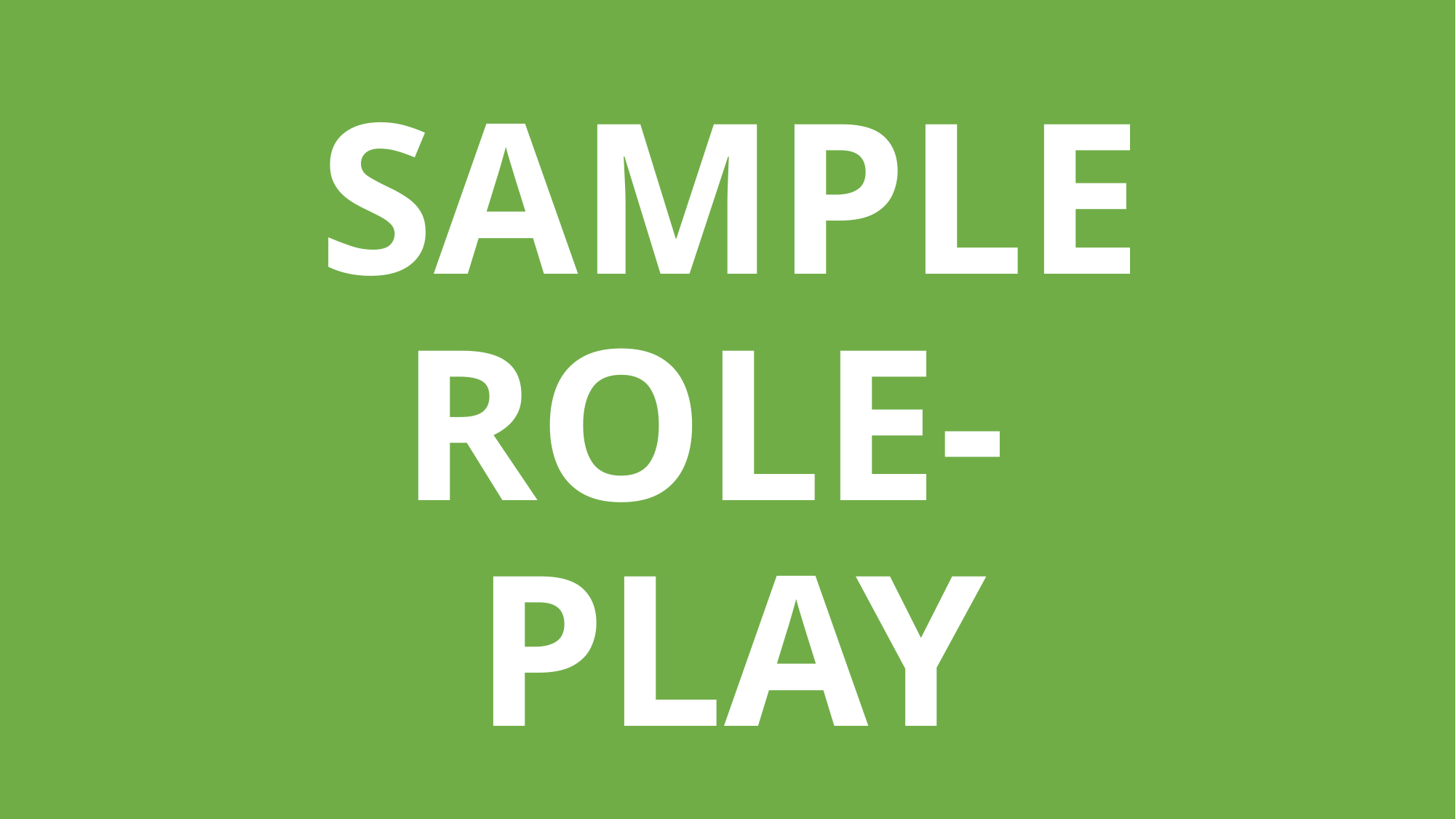

SAMPLE ROLE-
PLAY

## Slide 28
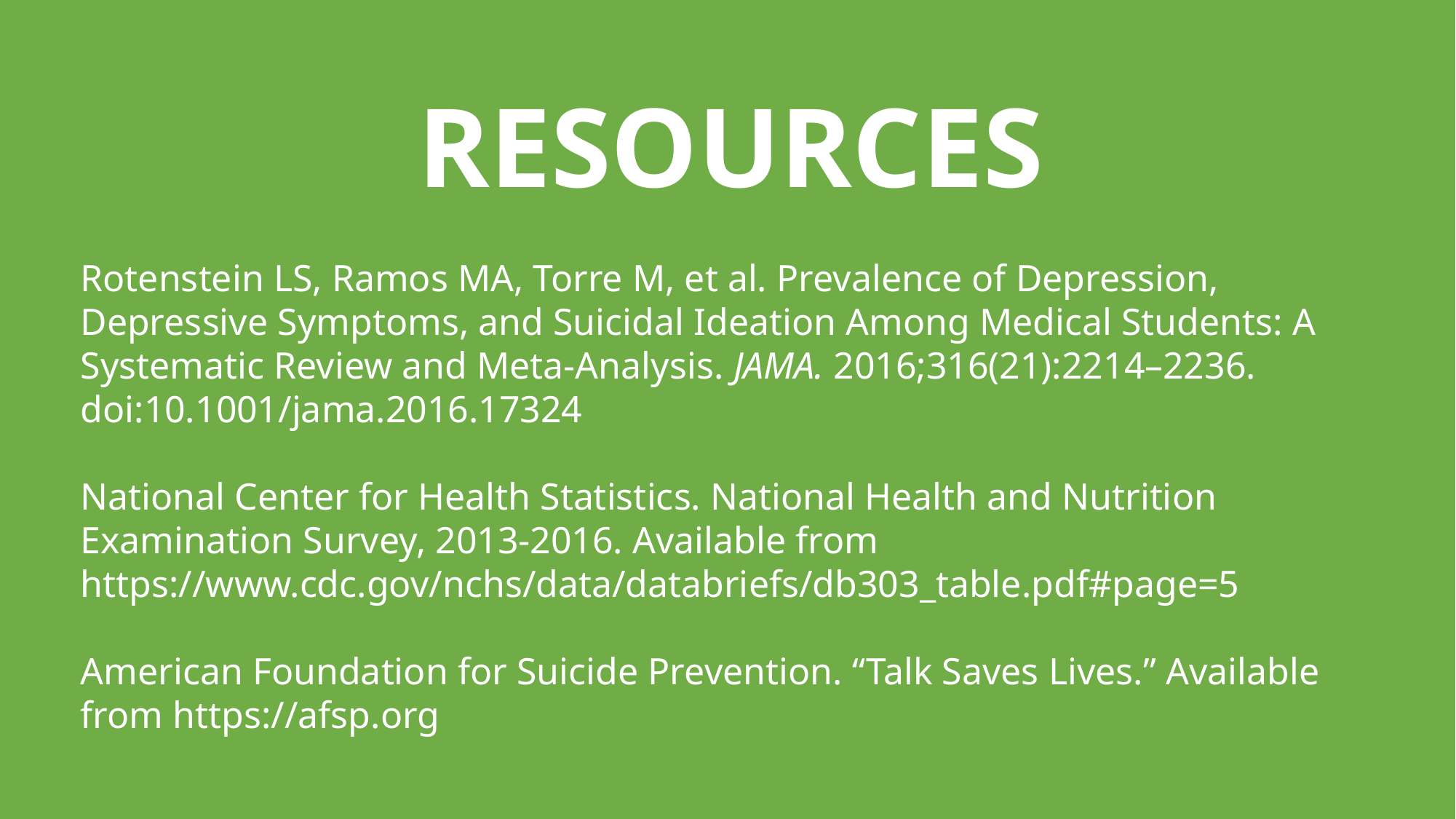

RESOURCES
Rotenstein LS, Ramos MA, Torre M, et al. Prevalence of Depression, Depressive Symptoms, and Suicidal Ideation Among Medical Students: A Systematic Review and Meta-Analysis. JAMA. 2016;316(21):2214–2236. doi:10.1001/jama.2016.17324
National Center for Health Statistics. National Health and Nutrition Examination Survey, 2013-2016. Available from https://www.cdc.gov/nchs/data/databriefs/db303_table.pdf#page=5
American Foundation for Suicide Prevention. “Talk Saves Lives.” Available from https://afsp.org

## Slide 29
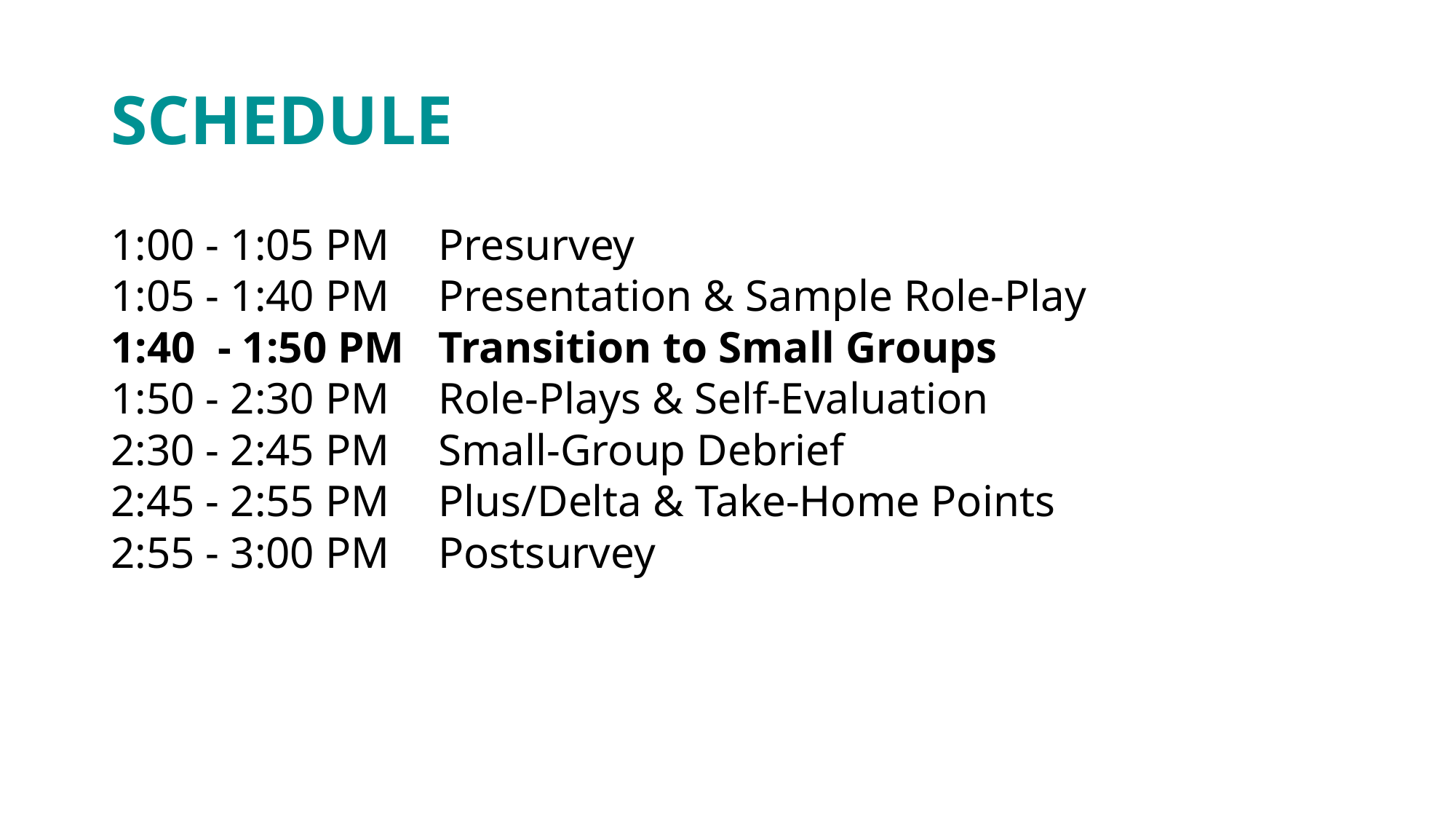

# SCHEDULE
1:00 - 1:05 PM	Presurvey
1:05 - 1:40 PM	Presentation & Sample Role-Play
1:40 - 1:50 PM	Transition to Small Groups
1:50 - 2:30 PM	Role-Plays & Self-Evaluation
2:30 - 2:45 PM	Small-Group Debrief
2:45 - 2:55 PM	Plus/Delta & Take-Home Points
2:55 - 3:00 PM	Postsurvey

## Slide 30
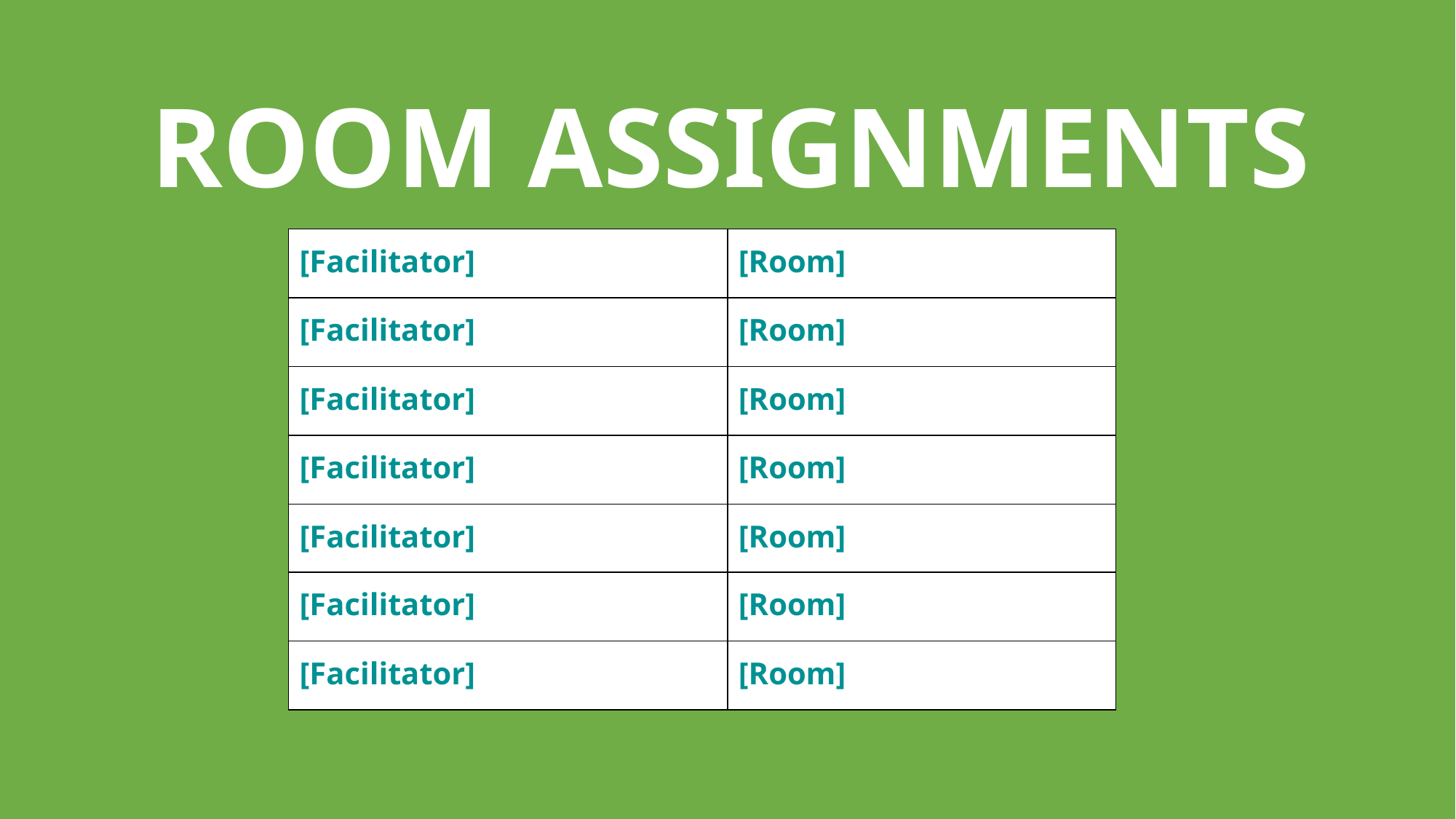

ROOM ASSIGNMENTS
| [Facilitator] | [Room] |
| --- | --- |
| [Facilitator] | [Room] |
| [Facilitator] | [Room] |
| [Facilitator] | [Room] |
| [Facilitator] | [Room] |
| [Facilitator] | [Room] |
| [Facilitator] | [Room] |
