# Supplementary material for: A Peer-to-Peer Suicide Prevention Workshop for Medical Students
Source: MedEdPORTAL. 2022 Apr 19;18:11241. doi: 10.15766/mep_2374-8265.11241 (PMC9016109; doi:10.15766/mep_2374-8265.11241)
Supplement: Supplementary file 1 — Didactic Slide Deck.pptxStudent Guide.docxFaculty Facilitation Guide.docxPre- and Postsurveys.docx [file mep_2374-8265.11241-s001.zip › D. Pre- and Postsurveys.docx]

Peer-to-Peer Suicide Prevention Training for Medical Students Impact Presurvey

We appreciate your time with completing this survey to support our broader mission in medical student education and suicide prevention training. In order to inform current and future training needs, we ask that you please complete this short survey regarding your experiences. Note that all responses are anonymous.

Please rate the following items on a scale from "1" Strongly Disagree to "5" Strongly Agree.

|  | Strongly Disagree (1) | Disagree (2) | Neither Agree nor Disagree (3) | Agree (4) | Strongly Agree (5) |
| --- | --- | --- | --- | --- | --- |
| 1. I am aware of risk and interventions concerning suicide prevention. |  |  |  |  |  |
| 1. I know how to recognize warning signs of suicide. |  |  |  |  |  |
| 1. I am confident in my ability to ask someone about suicidal thoughts. |  |  |  |  |  |
| 1. I believe I could persuade someone to get help. |  |  |  |  |  |
| 1. I am aware of resources for how to get help for someone. |  |  |  |  |  |
|  |  |  |  |  |  |
|  | Strongly Disagree (1) | Disagree (2) | Neither Agree nor Disagree (3) | Agree (4) | Strongly Agree (5) |
| 1. If someone I knew was showing warning signs of suicide, I would directly raise the question of suicide with them. |  |  |  |  |  |
| 1. If someone told me they were thinking of suicide, I would intervene. |  |  |  |  |  |
| 1. I don’t think I can prevent someone from suicide. |  |  |  |  |  |
| 1. I don’t feel competent to help a person at risk of suicide. |  |  |  |  |  |

Please rate the following items on a scale from "1" Not At All Likely to "5" Very Likely.

|  | Not At All Likely (1) | Unlikely (2) | Somewhat Likely (3) | Likely (4) | Very Likely (5) |
| --- | --- | --- | --- | --- | --- |
| 1. If another student were at high risk of suicide (e.g. expressing intent, plan), I would accompany them to urgent care services (e.g. [blinded] during office hours, Emergency Department). |  |  |  |  |  |
| 1. How likely are you to ask peers directly about suicide in the future? |  |  |  |  |  |

STOP

Peer-To-Peer Suicide Prevention Training for Medical Students Impact Postsurvey

We appreciate your time with completing this survey to support our broader mission in medical student education and suicide prevention training. In order to inform current and future training needs, we ask that you please complete this short survey regarding your experiences. Note that all responses are anonymous.

|  |
| --- |

Please rate the following items on a scale from "1" Strongly Disagree to "5" Strongly Agree.

|  | Strongly Disagree (1) | Disagree (2) | Neither Agree nor Disagree (3) | Agree (4) | Strongly Agree (5) |
| --- | --- | --- | --- | --- | --- |
| 1. I am aware of risk and interventions concerning suicide prevention. |  |  |  |  |  |
| 1. I know how to recognize warning signs of suicide. |  |  |  |  |  |
| 1. I am confident in my ability to ask someone about suicidal thoughts. |  |  |  |  |  |
| 1. I believe I could persuade someone to get help. |  |  |  |  |  |
| 1. I am aware of resources for how to get help for someone. |  |  |  |  |  |
| 1. If someone I knew was showing warning signs of suicide, I would directly raise the question of suicide with them. |  |  |  |  |  |
|  |  |  |  |  |  |
|  |  |  |  |  |  |
|  | Strongly Disagree (1) | Disagree (2) | Neither Agree nor Disagree (3) | Agree (4) | Strongly Agree (5) |
| 1. If someone told me they were thinking of suicide, I would intervene. |  |  |  |  |  |
| 1. I don’t think I can prevent someone from suicide. |  |  |  |  |  |
| 1. I don’t feel competent to help a person at risk of suicide. |  |  |  |  |  |

Please rate the following item on a scale from "1" Not At All Likely to "5" Very Likely.

|  | Not At All Likely (1) | Unlikely (2) | Somewhat Likely (3) | Likely (4) | Very Likely (5) |
| --- | --- | --- | --- | --- | --- |
| 1. If another student were at high risk of suicide (e.g. expressing intent, plan), I would accompany them to urgent care services (e.g. [blinded] during office hours, Emergency Department). |  |  |  |  |  |
| 1. How likely are you to ask peers directly about suicide in the future? |  |  |  |  |  |

1. What aspects of this peer-to-peer training did you find most helpful?

________________________________________________________________

1. What aspects of this training did you find least helpful?

________________________________________________________________

1. What content areas would you like to see for future trainings?

________________________________________________________________

1. What other resources/supports would you like to see to support students with suicidal thoughts?

________________________________________________________________

1. Do you believe this training will help you in helping someone that is experiencing suicidal thoughts? Yes or No
2. I would recommend this training program to other students. Yes or No
